# Reliability of HIV rapid diagnostic tests for self-testing compared with testing by health-care workers: a systematic review and meta-analysis

**DOI:** 10.1016/S2352-3018(18)30044-4

**Published:** 2018-04-24

**Authors:** Carmen Figueroa, Cheryl Johnson, Nathan Ford, Anita Sands, Shona Dalal, Robyn Meurant, Irena Prat, Karin Hatzold, Willy Urassa, Rachel Baggaley

**Affiliations:** a Department of HIV, World Health Organization, Geneva, Switzerland; b Department of Essential Medicines and Health Products, World Health Organization, Geneva, Switzerland; c Clinical Research Department, London School of Hygiene & Tropical Medicine, London, UK; d PSI Zimbabwe, Harare, Zimbabwe

## Abstract

**Background:**

The ability of individuals to use HIV self-tests correctly is debated. To inform the 2016 WHO recommendation on HIV self-testing, we assessed the reliability and performance of HIV rapid diagnostic tests when used by self-testers.

**Methods:**

In this systematic review and meta-analysis, we searched PubMed, PopLine, and Embase, conference abstracts, and additional grey literature between Jan 1, 1995, and April 30, 2016, for observational and experimental studies reporting on HIV self-testing performance. We excluded studies evaluating home specimen collection because patients did not interpret their own test results. We extracted data independently, using standardised extraction forms. Outcomes of interest were agreement between self-testers and health-care workers, sensitivity, and specificity. We calculated κ to establish the level of agreement and pooled κ estimates using a random-effects model, by approach (directly assisted or unassisted) and type of specimen (blood or oral fluid). We examined heterogeneity with the *I*^2^ statistic.

**Findings:**

25 studies met inclusion criteria (22 to 5662 participants). Quality assessment with QUADAS-2 showed studies had low risk of bias and incomplete reporting in accordance with the STARD checklist. Raw proportion of agreement ranged from 85·4% to 100%, and reported κ ranged from fair (κ 0·277, p<0·001) to almost perfect (κ 0·99, n=25). Pooled κ suggested almost perfect agreement for both types of approaches (directly assisted 0·98, 95% CI 0·96–0·99 and unassisted 0·97, 0·96–0·98; *I*^2^=34·5%, 0–97·8). Excluding two outliers, sensitivity and specificity was higher for blood-based rapid diagnostic tests (4/16) compared with oral fluid rapid diagnostic tests (13/16). The most common error that affected test performance was incorrect specimen collection (oral swab or finger prick). Study limitations included the use of different reference standards and no disaggregation of results by individuals taking antiretrovirals.

**Interpretation:**

Self-testers can reliably and accurately do HIV rapid diagnostic tests, as compared with trained health-care workers. Errors in performance might be reduced through the improvement of rapid diagnostic tests for self-testing, particularly to make sample collection easier and to simplify instructions for use.

**Funding:**

The Bill & Melinda Gates Foundation and Unitaid.

## Introduction

Interest in HIV self-testing—an approach to increase access to HIV testing—has increased since 2014.^1^ As a discreet and convenient approach, HIV self-testing might be most useful in reaching people who are reluctant or unable to access existing HIV testing services because of concerns about privacy, stigma, discrimination, and, in some contexts, criminalisation. According to various studies,^2^, ^3^, ^4^, ^5^, ^6^, ^7^ HIV self-testing is highly acceptable among many different population groups, including those with low testing coverage and who report barriers to and low uptake of existing HIV testing services. Despite this, some policy makers and users have raised concerns that self-testers might not be able to do the test or interpret the test results correctly.^1^

We did a systematic review to assess the reliability and performance of HIV rapid diagnostic tests when used by self-testers, compared with health-care workers. Although previous reviews assessed the accuracy of rapid diagnostic tests for self-testing,^8^, ^9^ they primarily focused on sensitivity and specificity and did not consider the validity of the reference standard. Thus, we systematically measure and report test concordance between self-testers and health-care workers to account for imperfect reference standards to establish the reliability and performance of rapid diagnostic tests used for self-testing.

**Research in context**

**Evidence before this study**

To diagnose HIV, at least two or three tests, depending on the HIV prevalence among the population being tested, are needed. The validity of using a single test as a reference standard is imperfect. Previous systematic reviews focused on sensitivity and specificity of HIV rapid diagnostic tests for self-testing. An initial search of PubMed, for studies published from Jan 1, 1995, to Jan 26, 2016, with the search terms “HIV self-testing” and “review”, indicated that the validity of the reference standard has not been considered previously.

**Added value of this study**

To inform a WHO recommendation, we assessed the reliability and performance of HIV rapid diagnostic tests used by self-testers compared with health-care workers, by calculating statistics on test concordance to account for the imperfect reference standard. We included studies that used products designed for self-testing in diverse country settings. Previous reviews were done when HIV self-testing was emerging; these reviews primarily drew from US and European studies that used professional-use products or prototypes that have since been adapted for HIV self-testing.

**Implications of all the available evidence**

Self-testers could reliably and accurately do an HIV rapid diagnostic test, whether assistance was provided or not, when compared with a trained health-care worker. Errors in the test procedure might be reduced by refinement of the design of rapid diagnostic tests for self-testing, improvement of manufacturer labelling and instructions for use, and provision of additional support with instructional videos. Modifications should always be the responsibility of the manufacturer.

## Methods

### Search strategy and selection criteria

This systematic review and meta-analysis followed the PRISMA standards (appendix pp 1–2). We searched PubMed, PopLine, and Embase for studies published from Jan 1, 1995, to April 30, 2016. We also reviewed six electronic HIV/AIDS conference databases (ie, Conference on Retroviruses and Opportunistic Infections, International AIDS Conference, International AIDS Society, American Public Health Association, National HIV Prevention Conference, and the HIV Diagnostics Conference) for all available years (appendix p 3).

We searched for grey literature through Google Scholar (first 100 titles of 201 results). We screened bibliographies of included articles and purposely selected and contacted experts (ie, academic researchers with ongoing studies on HIV self-testing) to identify additional sources. We contacted authors of relevant studies (up to two attempts) to retrieve relevant study information. We placed no language, age, study type, or geographical limitations on the search.

We included studies reporting the performance of rapid diagnostic tests by self-testers and those reporting the concordance or the sensitivity and specificity of rapid diagnostic tests compared with the results of testing done by a health-care worker. Two reviewers (CF, CJ) screened records independently and resolved disagreements through discussion and consensus.

We defined HIV self-testing as a process where an individual collects his or her specimen, does a test, and interprets their own test result.^1^ In the directly assisted approach, self-testers received an in-person demonstration of how to do the test or to interpret the test result; in the unassisted approach, self-testers were provided only with manufacturers' instructions for use included in the kit. All self-testers, irrespective of type of approach used, could access or receive assistance over the phone, through the internet, or with additional instructions (eg, videos, animations, or diagrams).^10^ We did not consider HIV counselling, linkage to care, and referral information as part of HIV self-testing assistance.^10^ We considered observed studies when participants were directly observed or video recorded to evaluate their HIV self-testing performance.

We excluded studies reporting on home specimen collection, concordance or sensitivity and specificity of self-testing, or self-monitoring devices for conditions other than HIV.

We defined the testing strategy used to establish the reference result as any testing sequence used to identify HIV infection (appendix pp 4–7). We classified testing strategies as aligned or not aligned with WHO guidance on the basis of the 2015 Consolidated guidelines on HIV testing services.^11^

### Data analysis

We defined measures of concordance (inter-reader reliability) as the percentage agreement and Cohen's κ^12^ between the health-care worker and the self-tester.

We defined measurements of accuracy as specificity and sensitivity. HIV positivity among participants was based on the number of HIV-positive participants with known status or who received an HIV-positive diagnosis during the study. HIV positivity was then categorised as high (≥5%) or low (<5%).^11^

Given the imperfect or absent reference standards among studies, to evaluate performance of HIV rapid diagnostic tests used by self-testers we first assessed whether the result of the index and the reference test agreed or disagreed.^13^ We then calculated the raw estimates of sensitivity and specificity.

We extracted data for true reactive, true non-reactive, false reactive, and false non-reactive results to calculate κ and raw estimates of sensitivity and specificity, and explored the effect for oral fluid and blood-based rapid diagnostic tests separately, by type of assistance (direct assistance or no assistance) and type of observation. We calculated raw estimates of sensitivity and specificity with Meta-DiSc software;^14^ we did not consider invalid or indeterminate values to avoid skewness of results.

We determined quality of studies using the Standards for Reporting Studies of Diagnostic Accuracy (STARD) checklist,^15^ and the Quality Assessment of Diagnostic Accuracy Studies (QUADAS-2).^16^ We considered high risk of partial verification bias if more than 10% of study participants did not have their HIV test results and status confirmed, and if the selection of patients to receive the reference standard was not randomised. We considered a study to have a high risk of differential verification bias if more than 10% of patients received testing with a different reference standard. CF scored studies for quality in terms of risk of bias and concerns regarding applicability.

Given the high study variability and the inclusion of multiple reference standards, we pooled κ estimates using a random-effects model with the R package metaphor, version 3.4.4.^13^ We assessed heterogeneity by visual inspection of forest plots and calculation of the *I*^2^ statistic (>25–50% moderate).^17^

### Role of the funding source

The funder of the study had no role in study design, data collection, data analysis, data interpretation, writing of the report, or the decision to submit for publication. The corresponding author had full access to all study data and final responsibility for the decision to submit for publication.

## Results

**Figure 1:**
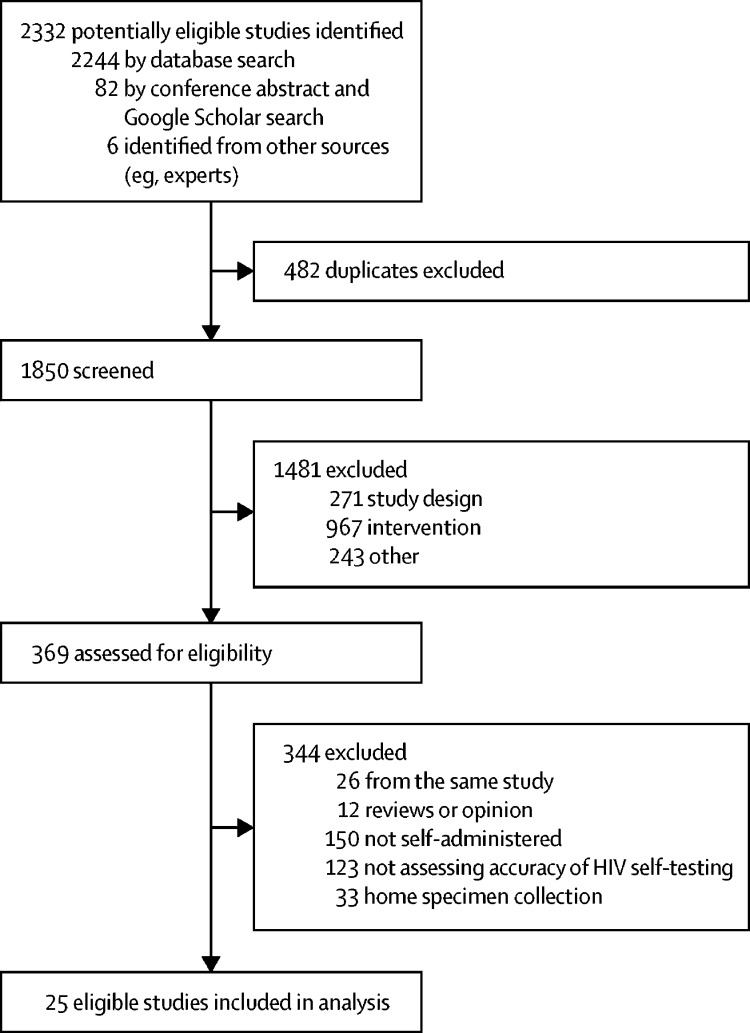
Study selection

After screening and removing duplicates, we included 25 studies^4^, ^6^, ^18^, ^19^, ^20^, ^21^, ^22^, ^23^, ^24^, ^25^, ^26^, ^27^, ^28^, ^29^, ^30^, ^31^, ^32^, ^33^, ^34^, ^35^, ^36^, ^37^, ^38^, ^39^, ^40^ in the review (figure 1). All studies^4^, ^6^, ^18^, ^19^, ^20^, ^21^, ^22^, ^23^, ^24^, ^25^, ^26^, ^27^, ^28^, ^29^, ^30^, ^31^, ^32^, ^33^, ^34^, ^35^, ^36^, ^37^, ^38^, ^39^, ^40^ evaluated concordance, 15 studies^4^, ^6^, ^18^, ^19^, ^20^, ^22^, ^26^, ^27^, ^28^, ^30^, ^31^, ^32^, ^34^, ^36^, ^38^ evaluated sensitivity and specificity, and one^25^ only evaluated sensitivity. 15 studies^4^, ^6^, ^18^, ^20^, ^24^, ^26^, ^28^, ^30^, ^31^, ^32^, ^33^, ^34^, ^35^, ^36^, ^38^ used oral fluid-based rapid diagnostic tests, six^21^, ^22^, ^25^, ^27^, ^37^, ^40^ used blood-based rapid diagnostic tests, and four^19^, ^23^, ^29^, ^39^ used both.

13 studies^4^, ^19^, ^22^, ^23^, ^24^, ^25^, ^26^, ^27^, ^28^, ^31^, ^32^, ^33^, ^39^ reported on unassisted HIV self-testing, 11^6^, ^18^, ^20^, ^29^, ^30^, ^34^, ^35^, ^36^, ^37^, ^38^, ^40^ reported on directly assisted HIV self-testing, and one^21^ reported on both approaches (table 1). 23 of 25 studies were observational in design (three cohort,^4^, ^6^, ^19^ 18 cross-sectional,^21^, ^22^, ^23^, ^24^, ^25^, ^26^, ^27^, ^28^, ^29^, ^32^, ^33^, ^34^, ^35^, ^36^, ^37^, ^38^, ^39^, ^40^ and two cross-sectional and qualitative^20^, ^30^), and two were randomised controlled trials.^18^, ^31^ Sample size varied from 22 to 5662 participants. HIV positivity among participants was available in 28 reports from 22 studies; 19 (68%) of 28 reports had a high HIV positivity,^4^, ^6^, ^18^, ^20^, ^22^, ^25^, ^26^, ^27^, ^28^, ^29^, ^30^, ^31^, ^32^, ^36^, ^39^ and eight (29%) had low HIV positivity.^4^, ^19^, ^23^, ^24^, ^33^, ^34^, ^35^, ^38^ Reference test strategy was not available in five of 25 studies, or was not aligned with WHO testing guidance in another five studies (appendix pp 4–7). 16 (64%) of 25 studies were considered to be at low risk of bias and applicability across all key domains for QUADAS-2 (appendix p 8). 17 (68%) of 25 studies also failed to fulfil at least 60% of the STARD criteria, with a mean of 16·2 available items out of 34 (appendix pp 9–34).

**Table 1:** Characteristics of included studies

	**Setting**	**Type of RDT specimen**	**Sample size**	**Male participants**	**Type of population**	**Age**	**Participants ever tested for HIV**	**Education**	**Study design**
**Directly assisted studies**									
Prazuck et al (2016)^37^	France, urban	Blood based	411*	54·5%	GP (100%)	..	78·6% (367/411)	..	Cross-sectional
Majam et al (2016)^40^	South Africa, urban	Blood based	60	46·7%	GP (100%)	..	..	33% primary, 34% secondary, 33% tertiary	Cross-sectional
MacGowan et al (2014)^29^	USA, urban	Both	22	100%	KP (100%)	..	..	45% (10/22) college graduate or higher, 41% (9/22) some college, 14% (3/22) less than college	Cross-sectional
Choko et al (2015)^6^	Malawi, urban	Oral fluid based	1649	..	GP (91·4%), PLHIV (8·5%)	..	..	..	Cohort
Choko et al (2011)^20^	Malawi, urban	Oral fluid based	283	48·1%	GP (92·7%), PLHIV (7·3%)	27 years (IQR 22–32)	62% (175/283)	40·3% (114/283) primary or less, 59·7% (169/283) higher than primary education	Cross-sectional and qualitative
Marley et al (2014)^30^	China, urban	Oral fluid based	229	..	GP (100%), VCT clients	..	..	..	Cross-sectional and qualitative
Martínez Pérez et al (2016)^36^	South Africa, rural	Oral fluid based	2198	33·7%	GP (84·7%), PLHIV (15·3%)	27·5 years (IQR 22–36)	94·1% (2068/2198)	..	Cross-sectional
Sarkar et al (2016)^38^	India, rural	Oral fluid based	202	0	Pregnant women (100%)	..	..	..	Cross-sectional
Pant Pai et al (2013)^34^	South Africa, urban	Oral fluid based	251	21·1%	HCW (100%)	..	86·8% (218/251)	59·8% (150/251) high school or less, 24·3% (61/251) college or technical school, 10·3% (26/251) university or higher, 4·8% (12/251) other	Cross-sectional
Pant Pai et al (2014)^35^	Canada, urban	Oral fluid based	145	38·6%	GP (100%)	22 years	49·4% (124/145)	College 20·6% (30/145), vocational or trade school 13·1% (19/145), university or higher 66·2% (96/145)	Cross-sectional
Asiimwe et al (2014; observed arm)^18^	Uganda, rural	Oral fluid based	123	62·6%	GP (100%)	27 years (IQR 22–32)	78·1% (96/123)	70·7% (87/123) less than primary, 21·1% (26/123) primary complete, 8·1% (10/123) secondary or higher	Randomised controlled trial
Asiimwe et al (2014; unobserved arm)^18^	Uganda, rural	Oral fluid based	123	52·1%	GP (100%)	28 years (IQR 23–32)	78·9% (97/123)	75·6% (93/123) less than primary, 13·8% (17/123) primary complete, 10·6% (13/123) secondary or higher	Randomised controlled trial
**Unassisted studies**									
Lee et al (2007)^27^	Singapore, urban	Blood based	350	89·4%	GP (74·9%), PLHIV (25·1%)	33 years (IQR 27–41)	74·8% (262/350)	12% (40/350) primary, 28% (97/350) secondary, 60% (210/350) at least tertiary education	Cross-sectional
Gras et al (2014)^25^	France, urban	Blood based	40	75·0%	PLHIV (100%)	..	..	32·5% (13/40) primary, 35% (14/40) secondary, 32·5% (13/40) tertiary education	Cross-sectional
Dong et al (2014)^22^	South Africa, rural	Blood based	233	28·8%	GP (100%)	..	89·3% (208/233)	Less than high school 63·5% (148/233), high school 29·2% (68/233), some tertiary education 7·3% (17/233)	Cross-sectional
Chavez et al (2016; oral fluid arm)^19^	USA, urban	Both	818†	100%	..	27 years (range 18–54)	82% (671/818)	86% some college	Cohort
Gaydos et al (2011; oral fluid arm)^23^	USA, urban	Oral fluid based	433	41·3%	GP (100%)	38·5 years (12·7)	..	..	Cross-sectional
Gaydos et al (2011; blood-based arm)^23^	USA, urban	Blood based	45	42·2%	GP (100%)	37·2 years (13·0)	..	..	Cross-sectional
Spielberg et al (2003)^39^	USA, urban	Both	340	..	PLHIV (100%)	..	..	..	Cross-sectional
Gaydos et al (2013)^24^	USA, urban	Oral fluid based	467	40·4%	GP (100%)	41 years	..	..	Cross-sectional
Kurth et al (2016)^26^	Kenya, urban	Oral fluid based	239	67·4%	GP (100%)	35·9 years (9·7)	90·7% (217/239)	12·04 years of education (3·13)	Cross-sectional
Li et al (2016)^28^	China, urban	Oral fluid based	200	100%	KP (100%)	29·6 years (8·66)		10% (10/200) primary or less; 44·5% (89/200) secondary, 45·5% (91/200) tertiary education	Cross-sectional
Nour et al (2012)^33^	USA, urban	Oral fluid based	249	42·2%	GP (100%)	41 years	0 (0/249)‡	..	Cross-sectional
Mavedzenge et al (2015; urban arm)^31^	Zimbabwe, urban	Oral fluid based	172	47·0%	GP (91·1%), PLHIV (8·9%)	30 years (range 18–70)	80% (138/172)	..	Randomised controlled trial
Mavedzenge et al (2015; urban arm)^31^	Zimbabwe, rural	Oral fluid based	62	47·0%	GP (91·1%), PLHIV (8·9%)	29 years (range 18–70)	89% (55/62)	..	Randomised controlled trial
Ng et al (2012)^32^	Singapore, urban	Oral fluid based	994	88·5%	GP (63·7%), PLHIV (20%), KP (6·3%)	32·4 years (IQR 27·1–40·5)	..	32·8% (326/994) less than high school, 29·8% (296/994) high school, 37·4% (372/994) at least college	Cross-sectional
FDA phase 2b (2012; observed arm)^4^	USA, urban	Oral fluid based	1031	66·9%	GP (42·4%), PLHIV (51·3%) KP (6·3%)	..	..	19·1% (197/1031) low literate; 45·6% (470/1031) high school or less	Cohort
FDA phase 3 (2012; unobserved arm)^4^	USA, urban	Oral fluid based	5662§	51·3%	GP (86·9%), KP (13·1%)	..	..	Low literate 28·0% (1624/5662); high school or less 54·9% (3113/5662)	Cohort
**Directly assisted and unassisted studies**									
de la Fuente et al (2012; directly assisted arm)^21^	Spain, urban	Blood based	208	58·2%	GP (63·8%), KP (36·2%)	..	39·9% (83/208)	57·2% (119/208) at least university, 41·3% (86/208) less than university	Cross-sectional
de la Fuente et al (2012; unassisted arm)^21^	Spain, urban	Blood based	313	70·5%	GP (63·8%), KP (36·2%)	..	51·1% (160/313)	48·5% (150/313) at least university, 51·5% (159/313) less than university	Cross-sectional

Data are n, %, mean (SD), median (IQR), median, mean (range), or % (n/N). RDT=rapid diagnostic test. GP=general population. KP=key population (men who have sex with men, sex workers, people who inject drugs, transgender people, and people in prisons or closed settings). PLHIV=people living with HIV. VCT=voluntary counselling and testing. HCW=health-care worker.

* Study was divided into two substudies: 264 participants performed the self-test, and 147 participants interpreted contrived pictures.

† 515 participants had all three results (both self-tests and dried blood home collection), 622 reported the oral fluid-based result, 565 reported the blood-based result, and 548 had the dried blood spot cards processed.

‡ In the previous 6 months.

§ 163 participants had no self-test results.

Of the 25 studies evaluating concordance between the result of an HIV rapid diagnostic test used by a self-tester compared with a result obtained by a health-care worker, 18^4^, ^6^, ^18^, ^19^, ^20^, ^21^, ^22^, ^23^, ^24^, ^28^, ^29^, ^31^, ^33^, ^34^, ^35^, ^37^, ^39^, ^40^ reported raw percentage of agreement, three^26^, ^27^, ^32^ reported a κ statistic, and four^23^, ^30^, ^36^, ^38^ reported both (table 2).

**Table 2:** HIVST concordance, reasons for disagreement, and errors in performance among studies (n=25)

	HIVST concordance*	**Reasons for disagreement**†	**HIV positivity**	**Type of observation**	**Errors in performance**	**Invalid results (invalid result/tests performed)**	**Reasons for invalid result**
**Directly assisted studies**							
Prazuck et al (2016)‡^37^	97·1% (142/147)	Non-reactive as reactive 2·7% (4/147), invalid as reactive 2·7% (4/147) or non-reactive 2·7% (4/147), non-reactive as invalid 1·4% (2/147)	..	Observed	..	1% (2/264)	..
Majam et al (2016)^40^	88% (53/60)	Non-reactive as reactive 1·7% (1/60), non-reactive as invalid 1·7% (1/60), reactive as non-reactive 1·7% (1/60), invalid 6·7% (4/60) as reactive or non-reactive	..	Observed	20 participants made mistakes; common errors were with blood collection and transferring and use of buffer	..	..
MacGowan et al (2014; oral fluid arm)^29^	95% (21/22)	Reactive as non-reactive 4·5% (1/22)	22·7% (5/22)	Observed	13·6% (3/22) participants made mistakes, common errors were spilling buffer and incorrect time to read the results	4·5% (1/22)	..
MacGowan et al (2014; blood-based arm)^29^	95% (20/21)	One HIV-positive participant with an invalid result interpreted his result as reactive 4·8% (1/21)	19% (4/21)		23·8% (5/21) participants made mistakes; common errors were incorrectly pushing the device into test holder and incorrect timing to read the results; one participant broke the device	9·5% (2/21)	Operational error
Choko et al (2015)§^6^	99·4%, 98·9%–99·7% (1639/1649)	Reactive as non-reactive 0·5% (9/1649), non-reactive as reactive 0·06% (1/1649)	8·6% (141/1649)	Observed	..	..	..
Choko et al (2011)¶^20^	99·2%, 97–100% (256/258)‖	One HIV-positive participant with a faint reactive result interpreted his result as uncertain, one HIV-positive participant had an invalid result	16·9% (48/283)	Non-observed	Common errors were touching collection pad, incorrect or incomplete swabbing, removing kit from developer too early, buffer spills, reading incorrectly, and fumbling vial or cap when opening developer fluid	0·4% (1/260)	..
Marley et al (2014)^30^	93·9% (215/229), κ 0·551, p=0·012	Reactive as invalid 3·1% (7/229), non-reactive as reactive 1·3% (3/229), invalid as non-reactive 1·3% (3/229), non-reactive as invalid 0·4% (1/229)	5·6% (13/229)	Observed	Common errors were unpreparedness before start 42% (94/229), inability to swab correctly 10% (23/229), buffer 15·9% (36/229), testing and reading test results 7·5% (17/229)	3·5% (8/229)	Six participants used test paper incorrectly
Martínez Pérez et al (2016)^36^	99·4% (2184/2198), κ 0·99**	Reactive as non-reactive 0·2% (4/2181)	15·3% (337/2198)	Observed	Two participants had to repeat the self-test, they accidentally spilled buffer vial; excluding known people living with HIV, 0·18% (4/2181) interpreted their tests as negative whereas the HCW interpreted the result as positive	0·5% (11/2198)	..
Sarkar et al (2016)^38^	98%, κ 0·566, p<0·001	Invalid as non-reactive 0·5% (1/202), non-reactive as invalid 0·9% (2/202)	0·9% (2/202)	Observed	..	0·9% (2/202)	..
Pant Pai (2013)^34^	98·8% (248/251)	Reactive as non-reactive 1·2% (3/251), two of which had a faint reactive line	3·6% (9/251)	Non-observed	Errors were in conducting and interpreting results	..	..
Pant Pai et al (2014)^35^	100% (145/145)	No difference between self-tester and HCW interpretation	0	Non-observed	..	..	..
Asiimwe et al (2014; observed arm)^18^	99·2% (122/123)	Non-reactive as invalid 0·8% (1/123)	10·6% (13/123)	Observed	19·5% (24/123) participants made mistakes; common errors were incorrect swabbing of gums, touching the collection pad and buffer spills	0·8% (1/123)	..
Asiimwe et al (2014; unobserved arm)^18^	94·3% (116/123)	..	16·3% (20/123)	Non-observed	No errors were reported	0·8% (1/117)††	
**Unassisted studies**							
Lee et al (2007)^27^	κ 0·277, p<0·001	Invalid as non-reactive 50·1% (176/350), invalid as reactive 4·6% (16/350) and reactive as non-reactive 0·3% (1/350)	25% (88/350)	Observed	..	56·3% (197/350)	85% failed to perform all steps correctly
Gras et al (2014)^25^	100%	No difference between self-tester and HCW interpretation	100% (40/40)	Observed	Common errors were insufficient blood, wrong lancet utilisation, and mixing of samples	5·7% (2/35)	..
Dong et al (2014)^22^	98·7% (230/233)	Reactive as non-reactive 0·5% (1/195), invalid as non-reactive 0·5% (1/195), non-reactive as invalid 0·5% (1/195)	18·9% (44/233)	Observed (video recorded)	..	0·4% (1/233)	..
Chavez et al (2016; oral fluid arm)‡‡^19^	98% (500/511)	Non-reactive as reactive 1·4%, non-reactive as invalid 0·8%	2% (11/622)	Non-observed	..	..	..
Chavez et al (2016; blood-based arm)‡‡^19^	99% (506/511)	Non-reactive as reactive 0·6%, non-reactive as invalid 0·4%	1% (7/565)			4·6% (26/565)	Operational error
Gaydos et al (2011)§§^23^	99·6%, 0·41–1·00 (476/478) weighted κ 0·75	Reactive as non-reactive 0·2% (1/478)	0·8% (4/478)	Observed	Difficulties were interpreting results, reading result chart, reading or following instructions, swabbing or pricking properly, or both, and opening the kit	0·2% (1/478)	Insufficient blood
Spielberg et al (2003; oral fluid arm)^39^	95%	..	100% (340/340)	Non-observed	Difficulties performing test decreased through changes made to instructions and labelling from 4·3% to 4%	4·1% (14/340)	Failure to put the test device in the vial with developer solution
Spielberg et a; (2003; blood-based arm)^39^	97%		100% (340/340)		Difficulties performing test decreased through changes made to instructions and labelling from 14% to 9%	7·9% (27/340)	
Gaydos et al (2013)^24^	100%	No difference between self-tester and HCW interpretation	0·2% (1/467)	Observed	..	..	..
Kurth et al (2016)^26^	κ 0·92 (0·84–0·99)	Non-reactive as invalid 12·5% (30/239), reactive as non-reactive 1·2% (3/239), non-reactive as reactive 0·4% (1/239)	14·6% (35/239)	Observed (video recorded)	Common errors were difficulty opening bottle, incorrect or incomplete swab of gums, and incorrect time to read the results; some individuals could have made multiple errors	15·1% (36/239)	All individuals recognised something went wrong with their test
Li et al (2016)^28^	95% (190/200)	Non-reactive as invalid 2·5% (5/200), reactive as non-reactive 1·5% (3/200), non-reactive as reactive 0·5% (1/200)	27·5% (55/200)	Observed	Common errors were incorrect or incomplete swab of gums, incorrect time to read the results, touching the collection pad, and buffer spills	3% (6/200)	..
Nour et al (2012)^33^	100%	No difference between self-tester and HCW interpretation	1·6% (4/249)	Observed	..	..	..
Mavedzenge et al (2015; urban arm)¶¶^31^	93% (160/172)	Non-reactive as invalid 2% (3/172)	9% (16/172)	Observed (video recorded)	Common errors were confusion with desiccant, buffer spills, dipping test device in developer before collecting sample, incorrect sampling, and incorrect time to read the results.	2·9% (5/172)	Participants with invalid results typically did not follow instructions
Mavedzenge et al (2015; rural arm)^31^	90% (56/62)	Non-reactive as reactive 4·8% (3/62)	8% (5/62)			3·2% (2/62)	
Ng et al (2012)^32^	κ 0·97, 0·95–0·99	Reactive as non-reactive 2·6% (5/983), reactive as invalid 0·5% (1/983), non-reactive as invalid 0·3% (2/983) and non-reactive as reactive 0·1% (1/983)	19·3% (192/994)	Observed	Common errors were incorrect or incomplete swab of gums, touching the collection pad during removal from packaging, or buffer spills	0·3% (3/983)	..
FDA phase 2b (2012; observed arm)‖‖^4^	93% (942/1013)	Reactive as non-reactive 0·9% (10/1013), non-reactive as reactive 0·1% (1/1013)	2·1% (120/5662)	Observed	Common errors were interpreting results (11/986), dipping device in developer prior to swabbing gums (11/986), buffer spills (4/986), incorrect swabbing (5/986), and could not find developer (2/986)	3·3% (33/986)	Operational errors
FDA phase 3 (2012; unobserved arm)***^4^	99·8% (5490/5499)	Reactive as non-reactive 0·1% (8/5499), non-reactive as reactive 0·01% (1/5499)	51% (526/1031)	Non-observed	Not understanding where to place the test stick after sample collection (1/4999)	0·6% (31/4999)	..
**Directly assisted and unassisted studies**							
de la Fuente et al (2012; directly assisted arm)^21^	85·4% (445/521)	Invalid as reactive 2·8%, non-reactive as reactive 2·7%, non-reactive as invalid 2%, reactive as invalid 1·9%, invalid as non-reactive 1·5% and reactive as non-reactive 1·1%	..	Observed	..	0·9% (2/208)	Most difficult step was obtaining blood and depositing it in the correct place
de la Fuente et al (2012; directly assisted arm)^21^	85·4% (445/521)	Invalid as reactive 2·8%, non-reactive as reactive 2·7%, non-reactive as invalid 2·1%, reactive as invalid 1·9%, invalid as non-reactive 1·5% and reactive as non-reactive 1·1%	..	Observed	..	0·9% (2/208)	Most difficult step was obtaining blood and depositing it in the correct place

Data are % (n/N); %, 95% CI, (n/N), κ, p value; or κ (95%CI). HIVST=HIV self-test. HCW=health-care worker. FDA=Food and Drug Administration.

* Reported as percentage of agreement or κ.

† Reason for disagreement assumes the self-tester perspective compared with the HCW.

‡ The study was divided into two substudies: 264 participants performed the self-test, and 147 participants interpreted contrived pictures.

§ Four participants were on antiretrovirals.

¶ 260 of 283 participants self-tested.

‖ Two participants had no confirmatory results.

** 17 known people living with HIV were not considered to calculate the κ.

†† Six participants had no results.

‡‡ 515 participants had all three results (both self-tests and dried blood home collection [dried blood spot]), 622 reported the oral fluid-based result, 565 reported the blood-based result, and 548 had the dried blood spot cards processed.

§§ Disaggregated results by type of specimen were not available. One participant was on antiretrovirals with undetectable viral load.

¶¶ One participant in the urban arm was on antiretrovirals.

‖‖ 1013 of 1031 participants completed the study.

*** 18 positives and 482 negatives were excluded from the accuracy analysis.

**Figure 2:**
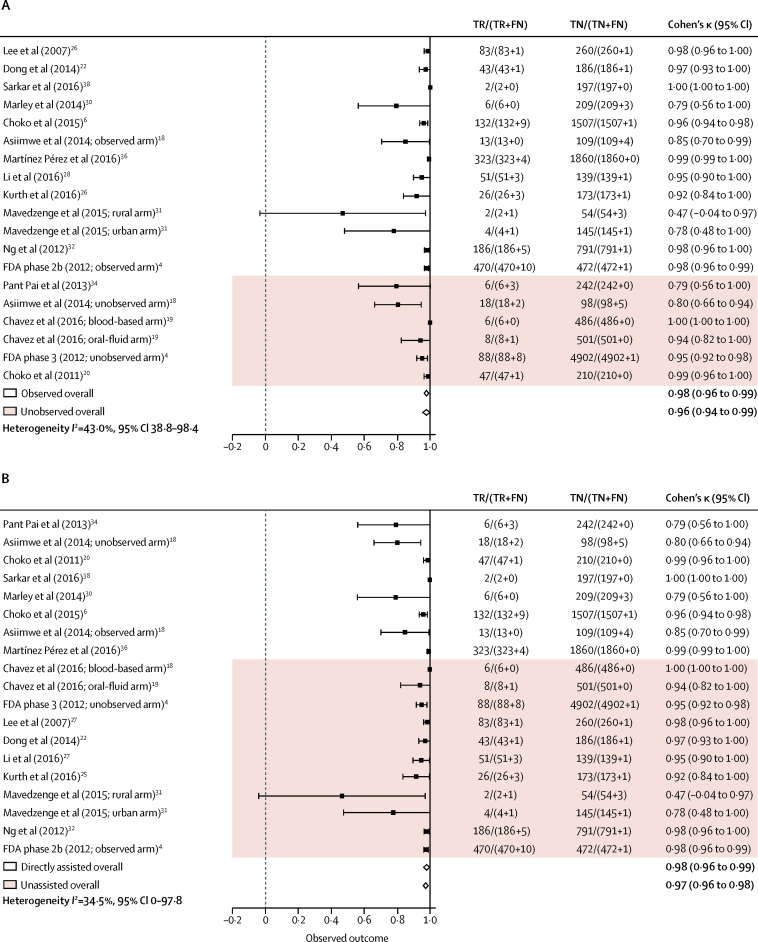
Cohen's κ across studies by method of observation (A) and type of approach (B) TR=true reactive result. FR=false reactive result. FN=false non-reactive result. TN=true non-reactive result.

Reported κ ranged from fair (κ 0·277, p<0·001) to almost perfect (κ 0·99).^23^, ^26^, ^27^, ^30^, ^32^, ^36^, ^38^ The raw proportion of agreement was high, ranging from 85·4% to 100%. Overall, our estimates of pooled agreement across studies were almost perfect for both types of approaches (directly assisted κ 0·98, 95% CI 0·96 to 0·99; unassisted κ 0·97, 0·96 to 0·98; *I*^2^=34·5%, 0 to 97·8; figure 2). Pooled estimates according to whether HIV self-testing was observed or not also had almost perfect agreement (observed 0·98, 0·96 to 0·99; unobserved 0·96, 0·94 to 0·99; *I*^2^=43·0%, 38·8 to 98·4; figure 2). The lowest estimated agreement (κ 0·47, −0·04 to 0·97) was in rural Zimbabwe; the study investigators attributed poor performance to low literacy in the population tested, and verbose instructions that needed further optimisation.^31^

The proportion of disagreements, assuming the self-tester perspective compared with health-care worker, ranged from 0% to 14·6%. Across 29 reports from 25 studies, four reports^24^, ^25^, ^33^, ^35^ found no difference in interpretation between self-testers and health-care workers. Most reported differences resulted from the interpretation of a reactive result as non-reactive (0·01–4·8%, 13 of 29 reports), a reactive result as invalid (2·7–6·7%, five of 29), a non-reactive result as reactive (0·1–4·5%, 14 of 29), an invalid result as reactive (0·5–3·1%, three of 29), an invalid result as non-reactive (0·3–12·5%, 13 of 29), or a non-reactive result as invalid (0·5–50%, seven of 29).

Reasons for disagreements were higher in directly assisted studies (2·7–6·7%) than unassisted studies (4·6%) when interpreting a reactive result as invalid, and were higher in unassisted studies (0·01–4·8%) than directly assisted studies (0·06–2·7%) when interpreting a reactive result as non-reactive.

Across 20 reports from 16 studies,^4^, ^6^, ^18^, ^19^, ^20^, ^22^, ^25^, ^26^, ^27^, ^28^, ^30^, ^31^, ^32^, ^34^, ^36^, ^38^ 16 (80%) of 20 reports had specificity of more than 98%. Sensitivity varied substantially; 18 (90%) of 20 reports had sensitivity of at least 80%. Two studies reported sensitivity of less than 80%: one^34^ had insufficient information on how to interpret faint positive lines, and the other^31^ suggested lengthy instructions were a barrier to participants in the rural arm, in which literacy levels were lower than the urban arm. Excluding these two studies,^31^, ^34^ sensitivity estimates were higher for blood-based rapid diagnostic tests (96·2–100%)^19^, ^22^, ^25^, ^27^ than oral fluid-based rapid diagnostic tests (80–100%),^4^, ^6^, ^18^, ^20^, ^26^, ^28^, ^30^, ^31^, ^32^, ^36^, ^38^ as were specificity estimates (blood-based 99·5–100% *vs* oral fluid 95·1–100%). Studies^4^, ^6^, ^18^, ^22^, ^25^, ^26^, ^27^, ^28^, ^30^, ^31^, ^32^, ^36^, ^38^ in which testing was observed reported a modest difference in sensitivity (80–100%) compared with unobserved studies^4^, ^18^, ^19^, ^20^, ^34^ (88·9–100%; table 3).

**Table 3:** Sensitivity and specificity of RDTs used for self-testing (n=16) by type of observation and approach

	**Sensitivity**	**TR/(TR+FN)**	**Specificity**	**TN/(TN+FR)**	**HIV positivity**	**Type of population**
**Unobserved studies**						
Pant Pai et al (2013)*^34^	66·7% (29·9–92·5)	6/(6+3)	100% (98·5–100)	242/(242+0)	3·6% (9/251)	HCW (100%)
Asiimwe et al (2014; unobserved arm)*^18^	90·0% (68·3–98·8)	18/(18+2)	95·1% (89·0–98·4)	98/(98+5)	17·2% (20/116)	GP (100%)
Chavez et al (2016; blood-based arm)†^19^	100% (54·1–100)	6/(6+0)	100% (99·2–100)	486/(486+0)	1·7% (9/515)	KP (100%)
Chavez et al (2016; oral fluid arm)*^19^	88·9% (51·8–99·7)	8/(8+1)	100% (99·3–100)	501/(501+0)	1·7% (9/515)	KP (100%)
FDA phase 3 (2012)*^4^	91·7% (84·2–96·3)	88/(88+8)	100% (99·9–100)	4902/(4902+1)	1·9% (96/4903)	GP (86·9%), KP (13·1%)
Choko et al (2011)*^20^	97·9% (88·9–99·9)	47/(47+1)	100% (98·3–100)	210/(210+0)	16·9% (48/283)	GP (100%)
**Observed studies**						
Gras et al (2014)†^25^	96·2% (80·4–99·9)	25/(25+1)	..	..	100% (26/26)	PLHIV (100%)
Lee (2007)†^27^	98·8% (93·5–100)	83/(83+1)	99·6% (97·9–100)	260/(260+1)	24·3% (84/345)	GP (90%), KP (10%)
Dong et al (2014)†^22^	97·7% (88·0–99·9)	43/(43+1)	99·5% (97·1–100)	186/(186+1)	19·0% (44/231)	GP (100%)
Sarkar et al (2016)*^38^	100% (15·8–100)	2/(2+0)	100% (98·1–100)	197/(197+0)	0·9% (2/202)	Pregnant women (100%)
Marley et al (2014)‡^30^	100% (54·1–100)	6/(6+0)	98·6% (95·9–99·7)	209/(209+3)	5·8% (13/222)	GP (100%), VCT clients
Choko et al (2015)§^6^	93·6% (88·2–97·0)	132/(132+9)	99·9% (99·6–100)	1507/(1507+1)	8·6% (141/1649)	GP (100%)
Asiimwe et al (2014; observed arm)*^18^	100% (75·3–100)	13/(13+0)	99·1% (95·0–100)	109/(109+4)	10·6% (13/122)	GP (100%)
Martínez Pérez et al (2016)*^36^	98·8% (96·9–99·7)	323/(323+4)	100% (99·8–100)	1860/(1860+0)	14·9% (327/2187)	GP (100%)
Li et al (2016)*^28^	94·4% (84·6–98·8)	51/(51+3)	99·3% (96·1–100)	139/(139+1)	28·9% (55/190)	KP (100%)
Kurth et al (2016)*^26^	89·7% (72·6–97·8)	26/(26+3)	99·4% (96·8–100)	173/(173+1)	14·3% (29/203)	GP (100%)
Mavedzenge et al (2015; rural arm)*^31^	66·7% (9·4–99·2)	2/(2+1)	94·7% (85·4–98·9)	54/(54+3)	8% (5/62)	GP (100%)
Mavedzenge et al (2015; urban arm)*‡^31^	80·0% (28·4–99·5)	4/(4+1)	97·8% (88·5–99·9)	145/(145+1)	9% (16/172)	GP (100%)
Ng et al (2012)*^32^	97·4% (94·0–99·1)	186/(186+5)	99·9% (99·3–100)	791/(791+1)	19·3% (192/994)	GP (63·7%), PLHIV (20%), KP (16·3%)
FDA phase 2b (2012)*^4^	97· 9% (96·2–99·0)	470/(470+10)	99·8% (98·8–100)	472/(472+1)	51·9% (526/1013)	GP (42·4%), PLHIV (513%), KP (6·3%)
**Directly assisted studies**						
Pant Pai et al (2013)*^34^	66·7% (29·9–92·5)	6/(6+3)	100% (98·5–100)	242/(242+0)	3·6% (9/251)	HCW (100%)
Sarkar et al (2016)*^38^	100% (15·8–100)	2/(2+0)	100% (98·1–100)	197/(197+0)	0·9% (2/202)	Pregnant women (100%)
Choko et al (2011)*^20^	97·9% (88·9–99·9)	47/(47+1)	100% (98·3–100)	210/(210+0)	16·9% (48/283)	GP (100%)
Choko et al (2015)*§^6^	93·6% (88·2–97·0)	132/(132+9)	99·9% (99·6–100)	1507/(1507+1)	8·6% (141/1649)	GP (100%)
Marley et al (2014)*‡^30^	100% (54·1–100)	6/(6+0)	98·6% (95·9–99·7)	209/(209+3)	5·8% (13/222)	GP (29%)
Asiimwe et al (2014; observed arm) *^18^	100% (75·3–100)	13/(13+0)	99·1% (95·0–100)	109/(109+4)	10·6% (13/122)	GP (100%)
Asiimwe et al (2014; unobserved arm)*^18^	90·0% (68·3–98·8)	18/(18+2)	95·1% (89·0–98·4)	98/(98+5)	17·2% (20/116)	GP (100%)
Martínez Pérez et al (2016)*^36^	98·8% (96·9–99·7)	323/(323+4)	100% (99·8–100)	1860/(1860+0)	14·9% (327/2187)	GP (100%)
**Unassisted studies**						
Gras et al (2014)†^25^	96·2% (80·4–99·9)	25/(25+1)	..	..	100% (26/26)	PLHIV (100%)
Lee et al (2007)†^27^	98·8% (93·5–100)	83/(83+1)	99·6% (97·9–100)	260/(260+1)	24·3% (84/345)	GP (90%), KP (10%)
Dong et al (2014)†^22^	97·7% (88·0–99·9)	43/(43+1)	99·5% (97·1–100)	186/(186+1)	19·0% (44/231)	GP (100%)
Chavez et al (2016; blood-based arm)†^19^	100% (54·1–100)	6/(6+0)	100% (99·2–100)	486/(486+0)	1·7% (9/515)	KP (100%)
Chavez et al (2016; oral fluid arm) *^19^	88·9% (51·8–99·7)	8/(8+1)	100% (99·3–100)	501/(501+0)	1·7% (9/515)	KP (100%)
Li et al (2016)*^28^	94·4% (84·6–98·8)	51/(51+3)	99·3% (96·1–100)	139/(139+1)	28·9% (55/190)	KP (100%)
Kurth et al (2016)*^26^	89·7% (72·6–97·8)	26/(26+3)	99·4% (96·8–100)	173/(173+1)	14·3% (29/203)	GP (100%)
FDA phase 3 (2012)*^4^	91·7% (84·2–96·3)	88/(88+8)	100% (99·9–100)	4902/(4902+1)	1·9% (96/4903)	GP (86·9%), KP (13·1%)
Mavedzenge et al (2015; rural arm)^31^	66·7% (9·4–99·2)	2/(2+1)	94·7% (85·4–98·9)	54/(54+3)	8% (5/62)	GP (100%)
Mavedzenge et al (2015; urban arm)*¶^31^	80·0% (28·4–99·5)	4/(4+1)	97·8% (88·5–99·9)	45/(45+1)	9% (16/172)	GP (100%)
Ng et al (2012)*^32^	97·4% (94·0–99·1)	186/(186+5)	99·9% (99·3–100)	791/(791+1)	19·3% (192/994)	GP (63·7%), PLHIV (20%), and KP (16·3%)
FDA phase 2b (2012)*^4^	97·9% (96·2–99·0)	470/(470+10)	99·8% (98·8–100)	472/(472+1)	51·9% (526/1013)	GP (42·4%), PLHIV (51·3%), and KP (6·3%)

Data are % (95% CI) or n/(n+n). TR=true reactive result. FR=false reactive result. FN=false non-reactive result. TN=true non-reactive result. HCW=health-care worker. GP=general population. KP=key population. FDA=US Food and Drug Administration. PLHIV=people living with HIV. VCT=voluntary counselling and testing

* Oral fluid-based.

† Finger stick-based or whole blood-based.

‡ This study assessed accuracy in a subsample of participants (229/800).

§ Four participants were on antiretrovirals; they tested negative via self-test and positive in confirmatory testing.

¶ One participant was on antiretrovirals; this person tested negative via self-test and positive in confirmatory testing. Heterogeneity: sensitivity *I*^2^ 55·1%; specificity *I*^2^ 78·7%. Spearman correlation coefficient −0·259, p 0·285.

A study^31^ from Zimbabwe with oral fluid-based rapid diagnostic tests, with data disaggregated by setting, found that urban populations had higher sensitivity (80%, 95% CI 28·4–99·5) than rural populations with lower literacy (66·7%, 9·4–99·2), and that this was also the case for specificity (urban 97·8%, 88·5–99·9 *vs* rural 94·7%, 85·4–98·9).

All studies^6^, ^18^, ^20^, ^30^, ^34^, ^36^, ^38^ addressing directly assisted HIV self-testing used oral fluid-based rapid diagnostic tests. The estimated sensitivity was similar to that in studies^4^, ^19^, ^26^, ^28^, ^31^, ^32^ with oral fluid rapid diagnostic tests within the unassisted approach (table 3).

Three studies included some participants taking antiretroviral drugs. In two studies,^6^, ^31^ participants had non-reactive test results, but later received confirmatory testing and were diagnosed or disclosed their HIV statuses afterward. In the third study,^23^ self-testers and health-care workers both obtained non-reactive results because they used the same oral test.

We identified 25 reports from 20 studies with information on invalid results: seven reports^20^, ^29^, ^30^, ^31^, ^32^, ^36^, ^40^ used the directly assisted approach, six^23^, ^25^, ^26^, ^28^, ^34^, ^39^ used the unassisted approach and two^4^, ^18^ used both approaches. Invalid results ranged from one (0·2%) of 478 tests to 197 (56·3%) of 350 tests (table 2).^4^, ^18^, ^19^, ^20^, ^21^, ^22^, ^23^, ^25^, ^26^, ^27^, ^28^, ^29^, ^30^, ^31^, ^32^, ^36^, ^37^, ^38^, ^39^

Although most participants were able to obtain a correct result, user errors among self-testers were noted in 15 reports. Of these reports, two found a high proportion of user error: one^27^ reported most users were unable to take blood samples or transfer blood specimens correctly (197 invalid results from 350 tests; 56·3%), and the other^26^ reported users were aware of making mistakes (36 invalid results from 239 tests; 15·1%). Excluding these studies, the proportion of invalid results was similar in studies^20^, ^29^, ^30^, ^31^, ^32^, ^36^, ^37^, ^38^, ^40^ of the directly assisted approach (0·3–9·5%) and studies^19^, ^22^, ^23^, ^25^, ^28^, ^39^ of the unassisted approach (0·2–7·9%). The proportion of invalid results was higher in studies^4^, ^18^, ^21^, ^22^, ^23^, ^25^, ^28^, ^29^, ^30^, ^31^, ^32^, ^36^, ^37^, ^38^ in which testing was observed (0·2–9·5%) when compared with unobserved studies^4^, ^18^, ^19^, ^20^, ^39^ (0·4–7·9%).

The proportion of studies reporting invalid results among self-testers was greater in studies^19^, ^21^, ^22^, ^25^, ^27^, ^29^, ^37^, ^39^, ^40^ using blood-based rapid diagnostic tests (0·4–9·5%) than studies^4^, ^18^, ^20^, ^23^, ^26^, ^28^, ^30^, ^31^, ^32^, ^36^, ^38^ using oral fluid-based rapid diagnostic tests (0·2–4·5%). Excluding studies^21^, ^25^, ^26^, ^27^, ^29^, ^39^ with feasibility of less than 60%, the proportion of invalid results was less than 5% (0·2–4·6%), regardless of the approach or specimen.

User errors described in studies of the directly assisted approach were incorrect or incomplete specimen collection (finger prick or oral swab),^20^, ^21^, ^30^, ^31^, ^40^ incorrect use or spillage of buffer,^20^, ^29^, ^30^, ^31^, ^36^, ^40^ incorrect transfer of blood specimen, and problems with the interpretation of results.^4^, ^20^, ^23^, ^30^, ^34^, ^39^, ^40^ Reported errors in studies of the unassisted approach included specimen collection (finger prick or oral swab),^23^, ^26^, ^28^ misinterpretation of test results,^23^, ^34^ incorrect time to read the results,^26^, ^28^ test kit opened incorrectly,^23^, ^26^ incorrect use or spillage of buffer,^28^ instructions not followed or read,^23^ or incorrect transfer of the blood specimen.^25^

In general, reported errors in performance were similar by type of specimen; however, studies using oral fluid rapid diagnostic tests reported errors in the interpretation of test results and studies using blood-based rapid diagnostic tests reported errors in transfer of the blood specimen.

Two studies^4^, ^25^ found that people with known HIV status had a higher proportion of errors (ie, when collecting the specimen) when self-testing compared with people with unknown HIV status (0·8% *vs* 0·2%), whereas a third study^27^ found that known HIV-positive participants were more likely to do the test correctly.

## Discussion

Self-testers can achieve the same results as health-care workers when using HIV rapid diagnostic tests and diagnostic accuracy of rapid diagnostic tests for self-testing is high. Application of the estimated ranges of sensitivity (80–100%) and specificity (95·1–100%) to a hypothetical group of 100 000 people with 1% of HIV prevalence would result in 0–200 HIV-positive cases being missed, and 0–4851 HIV-negative individuals being misidentified with a reactive result, excluding two outliers.^31^, ^34^

This systematic review and meta-analysis suggests that in the hands of self-testers, the sensitivity and specificity of blood-based rapid diagnostic tests were higher than oral fluid rapid diagnostic tests, although fewer studies used blood-based rapid diagnostic tests. The reduced sensitivity is probably explained by the lower quantity of HIV antibodies in oral fluid compared with whole blood, as observed in professional-use assessments.^41^ Although blood-based rapid diagnostic tests might have the potential to deliver more accurate results, more invalid results might occur because the greatest number of user errors was related to standard procedures when capillary tubes and pipettes were used.

Most studies had a high HIV positivity among participants where tests are expected to have a higher positive-predictive value than lower prevalence populations. Furthermore, imperfect reference standards might also decrease the degree of accuracy. We found wide variability in sensitivity estimates, which could be explained by the use of adapted rapid diagnostic tests not specifically designed for self-test use,^21^, ^22^, ^23^, ^25^, ^27^, ^37^, ^39^, ^40^ or used test kits before approval by national regulatory authorities, in 23 of 25 included studies.^4^, ^19^, ^20^, ^24^, ^26^, ^28^, ^29^, ^30^, ^31^, ^32^, ^33^, ^34^, ^35^, ^36^, ^38^

When we excluded six studies^21^, ^25^, ^26^, ^27^, ^29^, ^39^ with low feasibility, the proportion of invalid results met the minimum acceptable criteria for rapid diagnostic test performance (<5%);^42^ however, we still found no significant differences in the proportion of invalid results by type of approach, suggesting that use of a rapid diagnostic tests for self-testing without assistance will not increase the possibility of obtaining an invalid result.

Most invalid results and errors in performance reported by studies included in this review related to user errors and manufacturing defects. These invalid results and errors can be mitigated with instructions for use because the complexity of the test procedure or the complexity of the instructions can increase the possibility of failure in performance and incorrect interpretation of a result.

Recommendations include use of simple and clear language and well designed pictorial instructions, especially for the steps related to specimen collection, buffer use, and interpretation of results;^43^ easily identifiable kit components; reduction of the volume of specimen needed to do the test; and intuitive single-step test kits with controlled and automatic specimen collection, transfer, and buffer use.

In some settings, instructions could be adapted and validated for the cultural context and for less-skilled users, including individuals with low literacy or visual impairments. This could include translation in local languages, clear and large print instructions for use, detailed images and descriptions, or electronic documents or audio instructions. To improve performance of less-skilled users, instructions for use could be coupled with in-person or video demonstrations on how to do the test and interpret the result.

Product labelling should clearly state that people with reactive or invalid test results should seek further testing at a health facility. The labelling should also include information on test limitations in detecting HIV infection during the window period, for people taking pre-exposure prophylaxis, or in people with a suppressed immune response, such as people on antiretroviral drugs. This is a crucial issue because reports show that people with HIV on antiretrovial therapy might be using HIV self-testing kits to check and reconfirm their HIV status, and could obtain a false-negative result.^44^, ^45^

Strengths of this study include completeness of the search strategy, explicit inclusion criteria, a systematic approach to data collection, and independent assessment of each included study. Among the limitations were that most included studies used oral fluid-based rapid diagnostic tests, and studies used different and imperfect reference standard tests to identify HIV-positive individuals. Most studies did not compare approaches or specimens head-to-head. Results were considered biased in studies where the reference test strategy was not aligned with WHO testing guidance. Our last search was done on April 30, 2016, and since then 11 studies reporting on HIV self-testing performance have been published,^46^, ^47^, ^48^, ^49^, ^50^, ^51^, ^52^, ^53^, ^54^, ^55^, ^56^ of which eight are abstracts. These studies reached the same conclusion as we did, reporting that most participants were able to do the self-test correctly, and, where reported, the raw proportion of agreement was also high, ranging from 84% to 99%.^45^, ^46^, ^48^, ^49^, ^55^ Six of 11 studies would not have met our inclusion criteria, and the four that did meet the inclusion criteria might not have influenced our findings. Furthermore, most studies used adapted test kits that were not specifically designed or packaged for self-testing, and in some studies participants did not interpret their own results, but interpreted contrived devices or pictures or photographs.

No study provided information on people recently or acutely infected with HIV, and no study disaggregated data by individuals taking antiretroviral drugs. Because little data were available, we could not explore the influence of HIV prevalence, type of reference used, or study design. Selection bias is likely because most studies carefully selected participants; some studies included only known HIV-positive individuals. We did not assess publication bias because analytical methods are not well suited for testing observational data.^57^ Finally, although most studies were judged to be at low risk of bias, concerns remained about studies with small samples and the extent to which the findings can be generalised.

In summary, self-testers can achieve a high level of agreement with the results obtained by a health-care worker when using an HIV rapid diagnostic test for self-testing, whether or not assistance was provided. Errors in performance of the test procedure might be reduced through improvement of the design of rapid diagnostic tests for self-testing, clearer product labels, inclusion of simple instructions for use, and provision of additional support, such as instructional videos.
