# Supplementary material for: Reliability of HIV rapid diagnostic tests for self-testing compared with testing by health-care workers: a systematic review and meta-analysis
Source: Lancet HIV. 2018 Apr 24;5(6):e277–90. doi: 10.1016/S2352-3018(18)30044-4 (PMC5986793; doi:10.1016/S2352-3018(18)30044-4)
Supplement: Supplementary appendix [file mmc1.pdf]

THE LANCET
HIV

Supplementary appendix

This appendix formed part of the original submission and has been peer reviewed. We post it as supplied by the authors.

Supplement to: Figueroa C, Johnson C, Ford N, et al. Reliability of HIV rapid diagnostic tests for self-testing compared with testing by health-care workers: a systematic review and meta-analysis. *Lancet HIV* 2018; published online April 24. http://dx.doi.org/10.1016/S2352-3018(18)30044-4.

Web Appendix

Appendix 1. PRISMA 2009 Checklist

Section/topic	#	Checklist item	Reported on page #
**TITLE**			
Title	1	Identify the report as a systematic review, meta-analysis, or both.	1
**ABSTRACT**			
Structured summary	2	Provide a structured summary including, as applicable: background; objectives; data sources; study eligibility criteria, participants, and interventions; study appraisal and synthesis methods; results; limitations; conclusions and implications of key findings; systematic review registration number.	abstract
**INTRODUCTION**			
Rationale	3	Describe the rationale for the review in the context of what is already known.	3
Objectives	4	Provide an explicit statement of questions being addressed with reference to participants, interventions, comparisons, outcomes, and study design (PICOS).	3
**METHODS**			
Protocol and registration	5	Indicate if a review protocol exists, if and where it can be accessed (e.g., Web address), and, if available, provide registration information including registration number.	n/a
Eligibility criteria	6	Specify study characteristics (e.g., PICOS, length of follow-up) and report characteristics (e.g., years considered, language, publication status) used as criteria for eligibility, giving rationale.	4
Information sources	7	Describe all information sources (e.g., databases with dates of coverage, contact with study authors to identify additional studies) in the search and date last searched.	3-4
Search	8	Present full electronic search strategy for at least one database, including any limits used, such that it could be repeated.	Appendix 2
Study selection	9	State the process for selecting studies (i.e., screening, eligibility, included in systematic review, and, if applicable, included in the meta-analysis).	3-4
Data collection process	10	Describe method of data extraction from reports (e.g., piloted forms, independently, in duplicate) and any processes for obtaining and confirming data from investigators.	5
Data items	11	List and define all variables for which data were sought (e.g., PICOS, funding sources) and any assumptions and simplifications made.	3-5
Risk of bias in individual studies	12	Describe methods used for assessing risk of bias of individual studies (including specification of whether this was done at the study or outcome level), and how this information is to be used in any data synthesis.	4-5
Summary measures	13	State the principal summary measures (e.g., risk ratio, difference in means).	4-5
Synthesis of results	14	Describe the methods of handling data and combining results of studies, if done, including measures of consistency (e.g., I^2) for each meta-analysis.	5
Risk of bias across studies	15	Specify any assessment of risk of bias that may affect the cumulative evidence (e.g., publication bias, selective reporting within studies).	n/a
Additional analyses	16	Describe methods of additional analyses (e.g., sensitivity or subgroup analyses, meta-regression), if done, indicating which were pre-specified.	n/a

Appendix 1. PRISMA 2009 Checklist

**RESULTS**			
Study selection	17	Give numbers of studies screened, assessed for eligibility, and included in the review, with reasons for exclusions at each stage, ideally with a flow diagram.	4-5, Figure 1
Study characteristics	18	For each study, present characteristics for which data were extracted (e.g., study size, PICOS, follow-up period) and provide the citations.	Table 1
**Section/topic**	**#**	**Checklist item**	**Reported on page #**
Risk of bias within studies	19	Present data on risk of bias of each study and, if available, any outcome level assessment (see item 12).	Appendix 4-5
Results of individual studies	20	For all outcomes considered (benefits or harms), present, for each study: (a) simple summary data for each intervention group (b) effect estimates and confidence intervals, ideally with a forest plot.	Figure 2a-b
Synthesis of results	21	Present the main results of the review. If meta-analyses are done, include for each, confidence intervals and measures of consistency.	Figure 2a-b
Risk of bias across studies	22	Present results of any assessment of risk of bias across studies (see Item 15).	n/a
Additional analysis	23	Give results of additional analyses, if done (e.g., sensitivity or subgroup analyses, meta-regression [see Item 16]).	n/a
**DISCUSSION**			
Summary of evidence	24	Summarize the main findings including the strength of evidence for each main outcome; consider their relevance to key groups (e.g., healthcare providers, users, and policy makers).	21-22
Limitations	25	Discuss limitations at study and outcome level (e.g., risk of bias), and at review-level (e.g., incomplete retrieval of identified research, reporting bias).	22-23
Conclusions	26	Provide a general interpretation of the results in the context of other evidence, and implications for future research.	23-24
**FUNDING**			
Funding	27	Describe sources of funding for the systematic review and other support (e.g., supply of data); role of funders for the systematic review.	Funding disclosure section

From: Moher D, Liberati A, Tetzlaff J, Altman DG, The PRISMA Group (2009). Preferred Reporting Items for Systematic Reviews and Meta-Analyses: The PRISMA Statement. PLoS Med 6(7): e1000097. doi:10.1371/journal.pmed1000097

For more information, visit: **www.prisma-statement.org**.

Appendix 2. Search strategy

Last search 30.April.2016

1. Pubmed search strategy

Filters: Publication date from 1995/01/01 to 2016/04/30
#1 “HIV Infections” [MeSH] OR “HIV”[MeSH] OR “hiv”[tw] OR “hiv-1”[tw] OR “hiv-2”[tw] OR “hiv1”[tw] OR “hiv2”[tw] OR hiv infect*[tw] OR “human immunodeficiency virus”[tw] OR “human immunedeficiency virus”[tw] OR “human immuno-deficiency virus”[tw] OR “human immune-deficiency virus”[tw] OR ((human immun*) AND (“deficiency virus”[tw])) OR “acquired immunodeficiency syndrome”[tw] OR “acquired immunedeficiency syndrome”[tw] OR “acquired immuno-deficiency syndrome”[tw] OR “acquired immune-deficiency syndrome”[tw] OR ((acquired immun*) AND (“deficiency syndrome”[tw])) OR "Sexually Transmitted Diseases, Viral"[MeSH:NoExp]

#2 “hiv self-testing”[All Fields] OR “hiv self-test” [All Fields] OR "hivst" [All Fields] OR home test*[tiab] OR rapid test*[tiab] OR home self test*[tiab] OR home-based self test*[tiab] OR "self test"[tiab] OR self testing [tiab] OR “home test” [tiab] OR home testing [tiab]

#3 "Animals"[Mesh] NOT ("Animals"[Mesh] AND "Humans"[Mesh])

#4 (#2 AND #1) NOT #3

2. EMBASE

#1 'human immunodeficiency virus infection'/exp OR 'human immunodeficiency virus'/exp OR hiv:ti OR hiv:ab OR 'hiv-1':ti OR 'hiv-1':ab OR 'hiv-2':ti OR 'hiv-2':ab OR 'human immunodeficiency virus':ti OR 'human immuno deficiency':ab OR 'human immuno-deficiency virus':ti OR 'human immuno-deficiency virus':ab OR 'human immunedeficiency virus':ti OR 'human immune deficiency virus':ab OR 'human immune-deficiency virus':ti OR 'human immune-deficiency virus':ab OR 'acquired immune-deficiency syndrome':ti OR 'acquired immune-deficiency syndrome':ab OR 'acquired immunedeficiency syndrome':ti OR 'acquired immunedeficiency syndrome':ab OR 'acquired immunodeficiency syndrome':ti OR 'acquired immunodeficiency syndrome':ab OR 'acquired immuno-deficiency syndrome':ti OR 'acquired immuno-deficiency syndrome':ab AND [humans]/lim

AND

#2 'self evaluation'/exp OR (‘hivst’ OR ‘hiv self-testing’ OR ‘hiv self-test’ OR ‘hiv home testing’ OR ‘hiv home test’ OR ‘hiv rapid test’ OR ‘hiv rapid testing’ OR ‘home self test’ OR ‘home self testing’ OR ‘home-based self test’ OR ‘home-based self testing’):ab,ti,de,ca AND (1995:py OR 1996:py OR 1997:py OR 1998:py OR 1999:py OR 2000:py OR 2001:py OR 2002:py OR 2003:py OR 2004:py OR 2005:py OR 2006:py OR 2007:py OR 2008:py OR 2009:py OR 2010:py OR 2011:py OR 2012:py OR 2013:py OR 2014:py OR 2015:py OR 2016:py) AND [humans]/lim

3. Popline

("HIV" OR "human immunodeficiency virus infection") AND
(“HIV self testing” OR "hiv home testing" OR "hiv home test" OR "hiv rapid test" OR "hiv rapid testing" OR "HIVST")

Appendix 3. RDTs used for HIV self-testing and reference standard testing strategy among included studies (n=25)

Author and year of publication	RDT used for HIV self-testing	Reference test procedure	Description of the reference standard (algorithms)	Confirmatory testing strategy aligned with WHO	% requested assistance	Description of the support provided
**Directly assisted**						
MacGowan 2014 (oral-fluid arm)[1]	OraQuick Advance HIV 1/2 (Orasure Technologies, Bethlehem, PA, USA)	HCW verified participant-interpreted result	OraQuick Advance HIV 1/2 (Orasure Technologies, Bethlehem, PA, USA)	No	n/a	Participants used the instructions included in the package, which included a study telephone number and they had the option to watch a video on how to perform the test. A timer and testing materials were provided. By calling the study telephone, a HCW would approach and act as if they were answering the question by telephone, except for one person who asked for assistance when pricking his finger.
MacGowan 2014 (blood-based arm)[1]	Sure Check HIV 1/2 (Chembio Diagnostic Systems, Medford, NY, USA)*		Sure Check HIV 1/2 (Chembio Diagnostic Systems, Medford, NY, USA)		4.7% (1/21)	
Choko 2015[2]	OraQuick Advance HIV 1/2 (Orasure Technologies, Bethlehem, PA, USA)	Retesting by a HCW	Determine HIV 1/2, Alere and Uni-Gold Recombigen HIV, Trinity Biotech, CD4 count measurement	Yes	n/a	Written and demonstrated instructions, pre and post-test counseling, and facilitated HIV care assessment were provided. Participants were asked to demonstrate understanding using a cotton bud and vial of water in place of the kit. Instructions-for-use were modified and included pictures. Participants could opt to test with or without assistance.
Choko 2011[3]	OraQuick Advance HIV 1/2 (Orasure Technologies, Bethlehem, PA, USA)*	Retesting by a HCW	Determine Abbot Laboratories and Unigold Trinity Biotech, with a third test SD Bioline HIV 1/2 Standard Diagnostics, Inc., together with repeat of all three in case of any discordance	Yes	9.2% (26/283)	A HCW demonstrated briefly and discuss the self-testing process before testing, participants performed and interpret the results guided by illustrated instructions. Participants could request additional assistance if needed. Pre and post-counseling and written referral into HIV care services were provided.
Majam 2016[4]	n/a*	Participants interpreted contrived pictures	n/a	n/a	n/a	Participants received a brief demonstration on how to use the test, then received the instructions for use, performed the test, but not interpreted the results. Participants could ask for assistance if needed
Marley 2014[5]	Aware HIV-1/2 OMT (Calypte Biotech Co, Ltd, Petchaboon, Thailand)*	Retesting by a HCW	HCW read same test. Participants were confirmed with ELISA combined with Western-Blot	Yes	17.5% (40/229)	Participants received pre-test counseling and were asked to perform HIVST following instructions-for-use included in the kit. Participants could ask for assistance if needed.
Martínez-Perez 2016[6]	OraQuick Advance HIV 1/2 (Orasure Technologies, Bethlehem, PA, USA)*	Retesting by a HCW	Determine Abbot Laboratories, Unigold Trinity Biotech, and if indeterminate result referred for further testing	Yes	n/a	A HCW demonstrated how to perform the test according to manufacturer's package instructions. Participants received pre and post-testing counseling, and were linked to HIV-care at their preferred clinic, after HIV diagnostic was confirmed.
Prazuck 2016[7]	Autotest VIH finger stick-whole blood HIV test, (AAZ-LMB, Cedex France)*	Participants interpreted contrived pictures	n/a	n/a	21.2% (56/264)	Printed instructions were available. Each participant received pre and post-counseling for confirmatory HIV testing. Participants had the option to receive assistance in accordance to the hotline procedure established in the French protocol.

Author and year of publication	RDT used for HIV self-testing	Reference test procedure	Description of the reference standard (algorithms)	Confirmatory testing strategy aligned with WHO	% requested assistance	Description of the support provided
Sarkar 2016[8]	OraQuick Advance HIV 1/2 (Orasure Technologies, Bethlehem, PA, USA)[*]	Retesting by a HCW	SD Bioline, CombAIDS and Pareekshak Triline	Yes	8-26.3%[*]	Participants received pre and post-test counseling and linkage to care, a HCW explained the self-testing procedure using a simplified version with pictorial representation, and no demonstration was given. Participants could ask for assistance if needed.
Pant Pai 2013[9]	OraQuick Advance HIV 1/2 (Orasure Technologies, Bethlehem, PA, USA)[*]	Retesting by a HCW	ELISA with p24 Antigen tests (Architect HIV Ag/Ab Combo, Abbott Laboratories, Wiesbaden, Germany) and Western Blot for positives	Yes	n/a	Participants received pre and post-test counseling and information on HIV care. Participants could choose between an internet HIVST application with instructions in video or pictures or a paper-based application, with assistance sought over the phone or face-to-face if desired. In the initial version of the application, interpreting faint positive lines as positives was not included in the instructions.
Pant Pai 2014[10]	OraQuick Advance HIV 1/2 (Orasure Technologies, Bethlehem, PA, USA)[*†]	Retesting by a HCW before self-testing	Parallel ELISA with p24 Antigen tests (Architect HIV Ag/Ab Combo, Abbott Laboratories, Wiesbaden, Germany) and Western Blot for positives	Yes	n/a	In a first visit participants received instructions to use the self-test, in the second visit they were provided a kit containing a timer and pictorial reference guide that outlined all steps for self-testing; test instructions were also available as video. Participants received pre and post-test counseling and they could ask for assistance if needed.
Asiimwe 2014 (observed arm)[11]<br><br>Asiimwe 2014 (unobserved arm)[11]	OraQuick Advance HIV 1/2 (Orasure Technologies, Bethlehem, PA, USA)	Retesting by a HCW	Determine Abbot Laboratories, STAT-PAK Chembio Diagnostic Systems Inc. and Unigold Trinity Biotech as tiebreaker. For quality control all positives and 10% of negative samples were retested with Western Blot and HIV p24 ELISA.	No	41.5% (51/123)<br><br>23.6% (29/123)	All participants received pre and post-test counseling and a brief demonstration of how to use HIVST, printed instructions were available, and instructions were briefly re-read in the local language (Runyankore). The HCW was available for assistance if needed (including asking a timer).
**<u>Both approaches</u>**						
de la Fuente(2012) directly assisted arm[12]<br><br>de la Fuente (2012) unassisted arm[12]	Determine HIV 1/2 Ag/Ab Combo (Alere Medical, Matsudo-shi, Japan)[*‡]	Participants interpreted contrived pictures	n/a	n/a	n/a<br><br>n/a	A HCW demonstrated briefly the self-testing process before testing, and observed and guided the whole process, without intervening. Participants received pre and post-test counseling, written and pictorial instructions to perform the test.<br>Participants received pre and post-test counseling, written and pictorial instructions to perform the test and were instructed to read them carefully. The HCW did not provide any explanation or assistance.

Author and year of publication	RDT used for HIV self-testing	Reference test procedure	Description of the reference standard (algorithms)	Confirmatory testing strategy aligned with WHO	Description of the support provided
**Unassisted**					
Lee 2007[13]	Determine HIV 1/2 Abbott Laboratories, Abbott Park, IL)[*‡]	Retesting and verifying participant interpreted result by a HCW	Determine HIV 1/2 Abbott Laboratories, Abbott Park, IL)	No	Participants received pre and post-test counseling by a HCW, instructions-for-use were a 14-step pictorial sheet and a 4-step pictorial for result interpretation in English and Mandarin.
Chavez 2016 (oral-fluid arm)[14]<br>Chavez 2016 (blood-based arm)[14]	OraQuick Advance HIV 1/2 (Orasure Technologies, Bethlehem, PA, USA)<br>Sure Check HIV 1/2 (Chembio Diagnostic Systems, Medford, NY, USA)[*]	Dried blood spot collection kit	Western blot, Avio HIV 1 Micro Elisa and GS HIV combo Ag/Ab EIA	Yes	Participants used the instructions included in the package, including a telephone support. Participants had the option to watch an online video on how to perform the test.
Dong 2014[15]	iCARE OneStep HIV 1/2 (JAL Innovation, Singapore)[*‡]	Retesting by a HCW	iCARE OneStep HIV 1/2 and ELISA	Yes	Participants received illustrated instructions, supported by a telephone hotline.[§]
Gaydos 2011[16]	OraQuick Advance HIV 1/2 (Orasure Technologies, Bethlehem, PA, USA) or Uni-gold Recombigen HIV-1/2 (Trinity Biotech, Wicklow, Ireland)[*]	Retesting by a HCW before self-testing	OraQuick Advance HIV 1/2 (Orasure Technologies, Bethlehem, PA, USA) or Uni-gold Recombigen HIV-1/2 (Trinity Biotech, Wicklow, Ireland) and Western blot	Yes	Participants performed the test and interpret the results without assistance. Participants were given large plasticized instruction templates.
Gaydos 2013[17]	OraQuick Advance HIV 1/2 (Orasure Technologies, Bethlehem, PA, USA)[*]	Retesting by a HCW	OraQuick Advance HIV 1/2 (Orasure Technologies, Bethlehem, PA, USA) and Western Blot	Yes	Participants were provided with a mobile touch-screen tabled with 3 screen overview of the self-testing process and a large plasticized card with simple step-by-step directions and diagrams.
Gras 2014[18]	INSTI HIV-1/HIV-2 Antibody Test, (bioLytical, Richmond, BC Canada)[*‡]	Known PLHIV	n/a	n/a	Participants performed HIVST following the instructions-for-use (a detailed notice) without assistance.
Kurth 2016[19]	OraQuick Advance HIV 1/2 (Orasure Technologies, Bethlehem, PA, USA)[*]	Retesting by a HCW	Parallel testing with OraQuick Advance HIV 1/2 (Orasure Technologies, Bethlehem, PA, USA), Alere Determine HIV 1/2, Alere Medical Co. and then Vironostika HIV Uni-Form II Ag/Ab bioMérieux	Yes	Participants performed HIVST in a private space using pictorial instructions both in English and Kiswahili adapted from the manufacturer, without assistance from HCW. Participants received post-test counseling and referral and linkage to care.
Li 2016[20]	Aware HIV-1/2 OMT (Calypte Biotech Co, Ltd, Petchaboon, Thailand)[*]	Retesting by a HCW	Confirmed in CDC laboratory[¶]	Yes	Participants performed HIVST without any guidance or help from the HCW.
Nour 2012[21]	OraQuick Advance HIV 1/2 (Orasure Technologies, Bethlehem, PA, USA)[*]	Retesting by a HCW	OraQuick Advance HIV 1/2 (Orasure Technologies, Bethlehem, PA, USA)	No	Participants were given large plasticized instruction templates as visual aid and were asked to perform and interpret the test without any assistance from the HCW.

Spielberg 2003 (oral-fluid arm)[22]	OraQuick Advance HIV 1/2 (Orasure Technologies, Bethlehem, PA, USA)*	Known PLHIV	n/a	n/a	Participants were provided with kits and instructions and asked to perform HIVST without training or assistance from the HCW.
Spielberg 2003 (blood-based arm)[22]	n/a				
Mavedzenge 2015[23]	OraQuick Advance HIV 1/2 (Orasure Technologies, Bethlehem, PA, USA)*	Retesting by a HCW	SD Bioline as first test. If positive confirmed with Determine, with Chembio as the tiebreaker. Confirmatory testing was done using rapid HIV testing according to national algorithm.	No	Participants were given the instructional materials and left alone to complete their self-test. Participants had access to pictorial instructions, instructional video and a helpline for self-testing support and referral.
Ng 2012[24]	OraQuick Advance HIV 1/2 (Orasure Technologies, Bethlehem, PA, USA)*	Retesting by a HCW	OraQuick Advance HIV 1/2 (Orasure Technologies, Bethlehem, PA, USA), EIA and Western blot	Yes	Participants performed HIVST guided by 11-step pictorial instructions designed by the study team, without assistance from the HCW. Non PLHIV participants received pre and post-test counseling.
FDA phase 2b 2012 (observed arm)[25]	OraQuick Advance HIV 1/2 (Orasure Technologies, Bethlehem, PA, USA)*	Retesting by a HCW	OraQuick Advance HIV 1/2 (Orasure Technologies, Bethlehem, PA, USA) and Western blot	Yes	Participants received English and Spanish written and pictorial instructions to perform the test, and a booklet with pre-test and post-test information; they could access a toll free number for support. Pre and post-test counseling was provided.
FDA phase 3 2012 (unobserved arm)[25]		Retesting by a HCW	Serum EIA and Western blot	Yes	Participants received English and Spanish written and pictorial instructions to perform the test, and a booklet with pre-test and post-test information; they could access a toll free number for support. Participants chose where to self-test. Pre and post-test counseling was provided.

***** Before national authorities approval; † 21.8% (44/202) received assistance preparing the test kit, 26.3% (94/202) received assistance taking the sample and doing the test, of which 15% (30/202) received assistance swabbing their gums; and 8% (16/202) received assistance reading the result; ‡ especially adapted for the study; **§** 35.2% (82/233) called the helpline and received assistance during self-testing; ¶ no information on the specific assays utilized

RDT: rapid diagnostic test, HCW: health-care worker, n/a: non available, HIVST: HIV self-testing

Appendix 4. Tabular presentation for QUADAS-2 results of included studies (n=25)

	RISK OF BIAS				APPLICABILITY CONCERNS		
	PATIENT SELECTION	INDEX TEST	REFERENCE STANDARD	FLOW AND TIMING	PATIENT SELECTION	INDEX TEST	REFERENCE STANDARD
Asiimwe 2014[11]	+	+	+	+	+	+	+
Chavez 2016[14]	-	+	+	+	+	+	+
Choko 2011[3]	+	+	+	+	+	+	+
Choko 2015[2]	+	+	+	+	+	+	+
de la Fuente 2012[12]	+	-	-	-	+	+	+
Dong 2014[15]	?	+	+	+	+	+	+
Gaydos 2011[16]	+	+	-	+	+	+	+
Gaydos 2013[17]	-	+	+	+	+	+	+
Gras 2014[18]	?	-	?	-	+	+	+
Kurth 2016[19]	+	+	+	-	+	+	+
Lee 2007[13]	+	+	-	-	+	+	+
Li 2016[20]	+	+	+	+	+	+	+
MacGowan 2014[1]	?	+	-	+	?	+	+
Majam[4]	+	?	?	?	+	+	?
Marley 2014[5]	?	+	?	-	+	+	+
Mavedzenge 2015[23]	+	+	+	+	+	+	+
Ng 2012[24]	+	-	?	?	+	+	+
Nour 2012[21]	+	+	-	+	+	+	+
Pant Pai 2014[10]	+	+	-	+	-	+	+
Pant Pai 2013[9]	-	+	+	+	+	+	+
Martinez-Perez 2016[6]	+	+	+	+	+	+	+
FDA phase 2b 2012[25]	+	+	+	+	+	+	+
FDA phase 3 2012[25]	+	+	+	+	+	+	+
Prazuck 2016[7]	+	+	+	+	+	+	+
Sarkar 2016[8]	-	+	+	+	+	+	+
Spielberg 2003[22]	?	-	?	?	-	+	+

Appendix 5. STARD checklist for included studies (n=25)

Section & Topic	No	Item	Page
		**Asiimwe 2014[11]**	
TITLE OR ABSTRACT	1	Identification as a study of diagnostic accuracy using at least one measure of accuracy (such as sensitivity, specificity, predictive values, or AUC)	1
ABSTRACT	2	Structured summary of study design, methods, results, and conclusions (for specific guidance, see)	1
INTRODUCTION	3	Scientific and clinical background, including the intended use and clinical role of the index test	1
	4	Study objectives and hypotheses	2
METHODS Study design	5	Whether data collection was planned before the index test and reference standard were performed (prospective study) or after (retrospective study)	2,3
Participants	6	Eligibility criteria	2
	7	On what basis potentially eligible participants were identified (such as symptoms, results from previous tests, inclusion in registry)	2
	8	Where and when potentially eligible participants were identified (setting, location and dates)	2
	9	Whether participants formed a consecutive, random or convenience series	2
Test methods	10a STARD for Abstracts	Index test, in sufficient detail to allow replication	2,3
	10b	Reference standard, in sufficient detail to allow replication	2
	11	Rationale for choosing the reference standard (if alternatives exist)	2
	12a	Definition of and rationale for test positivity cut-offs or result categories of the index test, distinguishing pre-specified from exploratory	3,4
	12b	Definition of and rationale for test positivity cut-offs or result categories of the reference standard, distinguishing pre-specified from exploratory	3,4
	13a	Whether clinical information and reference standard results were available to the performers/readers of the index test	3
	13b	Whether clinical information and index test results were available to the assessors of the reference standard	3
Analysis	14	Methods for estimating or comparing measures of diagnostic accuracy	3
	15	How indeterminate index test or reference standard results were handled	n/a
	16	How missing data on the index test and reference standard were handled	n/a
	17	Any analyses of variability in diagnostic accuracy, distinguishing pre-specified from exploratory	n/a
	18	Intended sample size and how it was determined	n/a
RESULTS Participants	19	Flow of participants, using a diagram	3
	20	Baseline demographic and clinical characteristics of participants	n/a
	21a	Distribution of severity of disease in those with the target condition	n/a
	21b	Distribution of alternative diagnoses in those without the target condition	n/a
	22	Time interval and any clinical interventions between index test and reference standard	n/a
Test results	23	Cross tabulation of the index test results (or their distribution) by the results of the reference standard	5
	24	Estimates of diagnostic accuracy and their precision (such as 95% confidence intervals)	5
	25	Any adverse events from performing the index test or the reference standard	n/a
DISCUSSION	26	Study limitations, including sources of potential bias, statistical uncertainty, and generalizability	7,8
	27	Implications for practice, including the intended use and clinical role of the index test	7,8
OTHER INFORMATION	28	Registration number and name of registry	n/a
	29	Where the full study protocol can be accessed	n/a
	30	Sources of funding and other support; role of funders	8

Section & Topic	No	Item	Page
		**Lee 2007**[13]	
TITLE OR ABSTRACT	1	Identification as a study of diagnostic accuracy using at least one measure of accuracy (such as sensitivity, specificity, predictive values, or AUC)	n/a
ABSTRACT	2	Structured summary of study design, methods, results, and conclusions (for specific guidance, see)	1
INTRODUCTION	3	Scientific and clinical background, including the intended use and clinical role of the index test	1
	4	Study objectives and hypotheses	1
METHODS Study design	5	Whether data collection was planned before the index test and reference standard were performed (prospective study) or after (retrospective study)	1,2
Participants	6	Eligibility criteria	1
	7	On what basis potentially eligible participants were identified (such as symptoms, results from previous tests, inclusion in registry)	1
	8	Where and when potentially eligible participants were identified (setting, location and dates)	n/a
	9	Whether participants formed a consecutive, random or convenience series	1
Test methods	10a STARD for Abstracts	Index test, in sufficient detail to allow replication	2
	10b	Reference standard, in sufficient detail to allow replication	n/a
	11	Rationale for choosing the reference standard (if alternatives exist)	n/a
	12a	Definition of and rationale for test positivity cut-offs or result categories of the index test, distinguishing pre-specified from exploratory	n/a
	12b	Definition of and rationale for test positivity cut-offs or result categories of the reference standard, distinguishing pre-specified from exploratory	n/a
	13a	Whether clinical information and reference standard results were available to the performers/readers of the index test	2
	13b	Whether clinical information and index test results were available to the assessors of the reference standard	2
Analysis	14	Methods for estimating or comparing measures of diagnostic accuracy	2
	15	How indeterminate index test or reference standard results were handled	n/a
	16	How missing data on the index test and reference standard were handled	n/a
	17	Any analyses of variability in diagnostic accuracy, distinguishing pre-specified from exploratory	n/a
	18	Intended sample size and how it was determined	2
RESULTS Participants	19	Flow of participants, using a diagram	n/a
	20	Baseline demographic and clinical characteristics of participants	3
	21a	Distribution of severity of disease in those with the target condition	3
	21b	Distribution of alternative diagnoses in those without the target condition	n/a
	22	Time interval and any clinical interventions between index test and reference standard	n/a
Test results	23	Cross tabulation of the index test results (or their distribution) by the results of the reference standard	4
	24	Estimates of diagnostic accuracy and their precision (such as 95% confidence intervals)	4
	25	Any adverse events from performing the index test or the reference standard	n/a
DISCUSSION	26	Study limitations, including sources of potential bias, statistical uncertainty, and generalizability	4,5
	27	Implications for practice, including the intended use and clinical role of the index test	5
OTHER INFORMATION	28	Registration number and name of registry	n/a
	29	Where the full study protocol can be accessed	n/a
	30	Sources of funding and other support; role of funders	1

Section & Topic	No	Item	Page
		**Marley 2014[5]**	
TITLE OR ABSTRACT	1	Identification as a study of diagnostic accuracy using at least one measure of accuracy (such as sensitivity, specificity, predictive values, or AUC)	n/a
ABSTRACT	2	Structured summary of study design, methods, results, and conclusions (for specific guidance, see)	1
INTRODUCTION	3	Scientific and clinical background, including the intended use and clinical role of the index test	1,2
	4	Study objectives and hypotheses	2
METHODS<br>Study design	5	Whether data collection was planned before the index test and reference standard were performed (prospective study) or after (retrospective study)	2
Participants	6	Eligibility criteria	2
	7	On what basis potentially eligible participants were identified (such as symptoms, results from previous tests, inclusion in registry)	2
	8	Where and when potentially eligible participants were identified (setting, location and dates)	2
	9	Whether participants formed a consecutive, random or convenience series	2
Test methods	10a STARD for Abstracts	Index test, in sufficient detail to allow replication	n/a
	10b	Reference standard, in sufficient detail to allow replication	n/a
	11	Rationale for choosing the reference standard (if alternatives exist)	n/a
	12a	Definition of and rationale for test positivity cut-offs or result categories of the index test, distinguishing pre-specified from exploratory	n/a
	12b	Definition of and rationale for test positivity cut-offs or result categories of the reference standard, distinguishing pre-specified from exploratory	n/a
	13a	Whether clinical information and reference standard results were available to the performers/readers of the index test	n/a
	13b	Whether clinical information and index test results were available to the assessors of the reference standard	n/a
Analysis	14	Methods for estimating or comparing measures of diagnostic accuracy	n/a
	15	How indeterminate index test or reference standard results were handled	n/a
	16	How missing data on the index test and reference standard were handled	n/a
	17	Any analyses of variability in diagnostic accuracy, distinguishing pre-specified from exploratory	n/a
	18	Intended sample size and how it was determined	n/a
RESULTS<br>Participants	19	Flow of participants, using a diagram	n/a
	20	Baseline demographic and clinical characteristics of participants	n/a
	21a	Distribution of severity of disease in those with the target condition	n/a
	21b	Distribution of alternative diagnoses in those without the target condition	n/a
	22	Time interval and any clinical interventions between index test and reference standard	n/a
Test results	23	Cross tabulation of the index test results (or their distribution) by the results of the reference standard	4
	24	Estimates of diagnostic accuracy and their precision (such as 95% confidence intervals)	n/a
	25	Any adverse events from performing the index test or the reference standard	n/a
DISCUSSION	26	Study limitations, including sources of potential bias, statistical uncertainty, and generalizability	5
	27	Implications for practice, including the intended use and clinical role of the index test	4
OTHER INFORMATION	28	Registration number and name of registry	n/a
	29	Where the full study protocol can be accessed	n/a
	30	Sources of funding and other support; role of funders	6

Section & Topic	No	Item	Page
		**Ng 2012[24]**	
TITLE OR ABSTRACT	1	Identification as a study of diagnostic accuracy using at least one measure of accuracy (such as sensitivity, specificity, predictive values, or AUC)	1
ABSTRACT	2	Structured summary of study design, methods, results, and conclusions (for specific guidance, see)	1
INTRODUCTION	3	Scientific and clinical background, including the intended use and clinical role of the index test	1,2
	4	Study objectives and hypotheses	1
METHODS Study design	5	Whether data collection was planned before the index test and reference standard were performed (prospective study) or after (retrospective study)	2
Participants	6	Eligibility criteria	2
	7	On what basis potentially eligible participants were identified (such as symptoms, results from previous tests, inclusion in registry)	2
	8	Where and when potentially eligible participants were identified (setting, location and dates)	2
	9	Whether participants formed a consecutive, random or convenience series	2
Test methods	10a STARD for Abstracts	Index test, in sufficient detail to allow replication	2
	10b	Reference standard, in sufficient detail to allow replication	2
	11	Rationale for choosing the reference standard (if alternatives exist)	n/a
	12a	Definition of and rationale for test positivity cut-offs or result categories of the index test, distinguishing pre-specified from exploratory	n/a
	12b	Definition of and rationale for test positivity cut-offs or result categories of the reference standard, distinguishing pre-specified from exploratory	n/a
	13a	Whether clinical information and reference standard results were available to the performers/readers of the index test	2,3
	13b	Whether clinical information and index test results were available to the assessors of the reference standard	2,3
Analysis	14	Methods for estimating or comparing measures of diagnostic accuracy	3
	15	How indeterminate index test or reference standard results were handled	3
	16	How missing data on the index test and reference standard were handled	3
	17	Any analyses of variability in diagnostic accuracy, distinguishing pre-specified from exploratory	n/a
	18	Intended sample size and how it was determined	n/a
RESULTS Participants	19	Flow of participants, using a diagram	n/a
	20	Baseline demographic and clinical characteristics of participants	4
	21a	Distribution of severity of disease in those with the target condition	n/a
	21b	Distribution of alternative diagnoses in those without the target condition	n/a
	22	Time interval and any clinical interventions between index test and reference standard	n/a
Test results	23	Cross tabulation of the index test results (or their distribution) by the results of the reference standard	4
	24	Estimates of diagnostic accuracy and their precision (such as 95% confidence intervals)	n/a
	25	Any adverse events from performing the index test or the reference standard	n/a
DISCUSSION	26	Study limitations, including sources of potential bias, statistical uncertainty, and generalizability	7
	27	Implications for practice, including the intended use and clinical role of the index test	6
OTHER INFORMATION	28	Registration number and name of registry	n/a
	29	Where the full study protocol can be accessed	n/a
	30	Sources of funding and other support; role of funders	1

Section & Topic	No	Item	Page
		**de la Fuente 2012[12]**	
TITLE OR ABSTRACT	1	Identification as a study of diagnostic accuracy using at least one measure of accuracy (such as sensitivity, specificity, predictive values, or AUC)	n/a
ABSTRACT	2	Structured summary of study design, methods, results, and conclusions (for specific guidance, see)	1
INTRODUCTION	3	Scientific and clinical background, including the intended use and clinical role of the index test	1,2
	4	Study objectives and hypotheses	2
METHODS<br>Study design	5	Whether data collection was planned before the index test and reference standard were performed (prospective study) or after (retrospective study)	2
Participants	6	Eligibility criteria	2
	7	On what basis potentially eligible participants were identified (such as symptoms, results from previous tests, inclusion in registry)	n/a
	8	Where and when potentially eligible participants were identified (setting, location and dates)	2
	9	Whether participants formed a consecutive, random or convenience series	2
Test methods	10a STARD for Abstracts	Index test, in sufficient detail to allow replication	2,3
	10b	Reference standard, in sufficient detail to allow replication	2,3
	11	Rationale for choosing the reference standard (if alternatives exist)	
	12a	Definition of and rationale for test positivity cut-offs or result categories of the index test, distinguishing pre-specified from exploratory	n/a
	12b	Definition of and rationale for test positivity cut-offs or result categories of the reference standard, distinguishing pre-specified from exploratory	n/a
	13a	Whether clinical information and reference standard results were available to the performers/readers of the index test	2
	13b	Whether clinical information and index test results were available to the assessors of the reference standard	2
Analysis	14	Methods for estimating or comparing measures of diagnostic accuracy	3
	15	How indeterminate index test or reference standard results were handled	3
	16	How missing data on the index test and reference standard were handled	n/a
	17	Any analyses of variability in diagnostic accuracy, distinguishing pre-specified from exploratory	n/a
	18	Intended sample size and how it was determined	n/a
RESULTS<br>Participants	19	Flow of participants, using a diagram	4
	20	Baseline demographic and clinical characteristics of participants	5
	21a	Distribution of severity of disease in those with the target condition	n/a
	21b	Distribution of alternative diagnoses in those without the target condition	n/a
	22	Time interval and any clinical interventions between index test and reference standard	n/a
Test results	23	Cross tabulation of the index test results (or their distribution) by the results of the reference standard	7
	24	Estimates of diagnostic accuracy and their precision (such as 95% confidence intervals)	n/a
	25	Any adverse events from performing the index test or the reference standard	3
DISCUSSION	26	Study limitations, including sources of potential bias, statistical uncertainty, and generalizability	
	27	Implications for practice, including the intended use and clinical role of the index test	9
OTHER INFORMATION	28	Registration number and name of registry	n/a
	29	Where the full study protocol can be accessed	n/a
	30	Sources of funding and other support; role of funders	1

Section & Topic	No	Item	Page
		**Gras 2014[18]**	
TITLE OR ABSTRACT	1	Identification as a study of diagnostic accuracy using at least one measure of accuracy (such as sensitivity, specificity, predictive values, or AUC)	n/a
ABSTRACT	2	Structured summary of study design, methods, results, and conclusions (for specific guidance, see)	1
INTRODUCTION	3	Scientific and clinical background, including the intended use and clinical role of the index test	1
	4	Study objectives and hypotheses	1
METHODS Study design	5	Whether data collection was planned before the index test and reference standard were performed (prospective study) or after (retrospective study)	1
Participants	6	Eligibility criteria	1
	7	On what basis potentially eligible participants were identified (such as symptoms, results from previous tests, inclusion in registry)	1
	8	Where and when potentially eligible participants were identified (setting, location and dates)	n/a
	9	Whether participants formed a consecutive, random or convenience series	1
Test methods	10a STARD for Abstracts	Index test, in sufficient detail to allow replication	n/a
	10b	Reference standard, in sufficient detail to allow replication	n/a
	11	Rationale for choosing the reference standard (if alternatives exist)	n/a
	12a	Definition of and rationale for test positivity cut-offs or result categories of the index test, distinguishing pre-specified from exploratory	n/a
	12b	Definition of and rationale for test positivity cut-offs or result categories of the reference standard, distinguishing pre-specified from exploratory	n/a
	13a	Whether clinical information and reference standard results were available to the performers/readers of the index test	n/a
	13b	Whether clinical information and index test results were available to the assessors of the reference standard	n/a
Analysis	14	Methods for estimating or comparing measures of diagnostic accuracy	n/a
	15	How indeterminate index test or reference standard results were handled	n/a
	16	How missing data on the index test and reference standard were handled	n/a
	17	Any analyses of variability in diagnostic accuracy, distinguishing pre-specified from exploratory	n/a
	18	Intended sample size and how it was determined	n/a
RESULTS Participants	19	Flow of participants, using a diagram	n/a
	20	Baseline demographic and clinical characteristics of participants	1
	21a	Distribution of severity of disease in those with the target condition	n/a
	21b	Distribution of alternative diagnoses in those without the target condition	n/a
	22	Time interval and any clinical interventions between index test and reference standard	n/a
Test results	23	Cross tabulation of the index test results (or their distribution) by the results of the reference standard	n/a
	24	Estimates of diagnostic accuracy and their precision (such as 95% confidence intervals)	n/a
	25	Any adverse events from performing the index test or the reference standard	n/a
DISCUSSION	26	Study limitations, including sources of potential bias, statistical uncertainty, and generalizability	n/a
	27	Implications for practice, including the intended use and clinical role of the index test	n/a
OTHER INFORMATION	28	Registration number and name of registry	n/a
	29	Where the full study protocol can be accessed	n/a
	30	Sources of funding and other support; role of funders	n/a

Section & Topic	No	Item	Page
		**Kurth 2016[19]**	
TITLE OR ABSTRACT	1	Identification as a study of diagnostic accuracy using at least one measure of accuracy (such as sensitivity, specificity, predictive values, or AUC)	1
ABSTRACT	2	Structured summary of study design, methods, results, and conclusions (for specific guidance, see)	1
INTRODUCTION	3	Scientific and clinical background, including the intended use and clinical role of the index test	1,2
	4	Study objectives and hypotheses	2
METHODS Study design	5	Whether data collection was planned before the index test and reference standard were performed (prospective study) or after (retrospective study)	2
Participants	6	Eligibility criteria	2
	7	On what basis potentially eligible participants were identified (such as symptoms, results from previous tests, inclusion in registry)	2
	8	Where and when potentially eligible participants were identified (setting, location and dates)	2
	9	Whether participants formed a consecutive, random or convenience series	2
Test methods	10a STARD for Abstracts	Index test, in sufficient detail to allow replication	2,3
	10b	Reference standard, in sufficient detail to allow replication	2,3
	11	Rationale for choosing the reference standard (if alternatives exist)	3
	12a	Definition of and rationale for test positivity cut-offs or result categories of the index test, distinguishing pre-specified from exploratory	n/a
	12b	Definition of and rationale for test positivity cut-offs or result categories of the reference standard, distinguishing pre-specified from exploratory	3
	13a	Whether clinical information and reference standard results were available to the performers/readers of the index test	n/a
	13b	Whether clinical information and index test results were available to the assessors of the reference standard	n/a
Analysis	14	Methods for estimating or comparing measures of diagnostic accuracy	3
	15	How indeterminate index test or reference standard results were handled	3
	16	How missing data on the index test and reference standard were handled	3
	17	Any analyses of variability in diagnostic accuracy, distinguishing pre-specified from exploratory	n/a
	18	Intended sample size and how it was determined	3
RESULTS Participants	19	Flow of participants, using a diagram	4
	20	Baseline demographic and clinical characteristics of participants	5
	21a	Distribution of severity of disease in those with the target condition	n/a
	21b	Distribution of alternative diagnoses in those without the target condition	n/a
	22	Time interval and any clinical interventions between index test and reference standard	n/a
Test results	23	Cross tabulation of the index test results (or their distribution) by the results of the reference standard	7
	24	Estimates of diagnostic accuracy and their precision (such as 95% confidence intervals)	4,5
	25	Any adverse events from performing the index test or the reference standard	n/a
DISCUSSION	26	Study limitations, including sources of potential bias, statistical uncertainty, and generalizability	8
	27	Implications for practice, including the intended use and clinical role of the index test	9
OTHER INFORMATION	28	Registration number and name of registry	n/a
	29	Where the full study protocol can be accessed	n/a
	30	Sources of funding and other support; role of funders	9

Section & Topic	No	Item	Page
		**Pant Pai 2014[10]**	
TITLE OR ABSTRACT	1	Identification as a study of diagnostic accuracy using at least one measure of accuracy (such as sensitivity, specificity, predictive values, or AUC)	n/a
ABSTRACT	2	Structured summary of study design, methods, results, and conclusions (for specific guidance, see)	1
INTRODUCTION	3	Scientific and clinical background, including the intended use and clinical role of the index test	1,2
	4	Study objectives and hypotheses	2
METHODS Study design	5	Whether data collection was planned before the index test and reference standard were performed (prospective study) or after (retrospective study)	2
Participants	6	Eligibility criteria	2
	7	On what basis potentially eligible participants were identified (such as symptoms, results from previous tests, inclusion in registry)	2
	8	Where and when potentially eligible participants were identified (setting, location and dates)	2
	9	Whether participants formed a consecutive, random or convenience series	2
Test methods	10a STARD for Abstracts	Index test, in sufficient detail to allow replication	3
	10b	Reference standard, in sufficient detail to allow replication	2
	11	Rationale for choosing the reference standard (if alternatives exist)	n/a
	12a	Definition of and rationale for test positivity cut-offs or result categories of the index test, distinguishing pre-specified from exploratory	n/a
	12b	Definition of and rationale for test positivity cut-offs or result categories of the reference standard, distinguishing pre-specified from exploratory	n/a
	13a	Whether clinical information and reference standard results were available to the performers/readers of the index test	2,3
	13b	Whether clinical information and index test results were available to the assessors of the reference standard	2,3
Analysis	14	Methods for estimating or comparing measures of diagnostic accuracy	3
	15	How indeterminate index test or reference standard results were handled	3
	16	How missing data on the index test and reference standard were handled	3
	17	Any analyses of variability in diagnostic accuracy, distinguishing pre-specified from exploratory	n/a
	18	Intended sample size and how it was determined	n/a
RESULTS Participants	19	Flow of participants, using a diagram	3
	20	Baseline demographic and clinical characteristics of participants	4
	21a	Distribution of severity of disease in those with the target condition	n/a
	21b	Distribution of alternative diagnoses in those without the target condition	n/a
	22	Time interval and any clinical interventions between index test and reference standard	n/a
Test results	23	Cross tabulation of the index test results (or their distribution) by the results of the reference standard	n/a
	24	Estimates of diagnostic accuracy and their precision (such as 95% confidence intervals)	n/a
	25	Any adverse events from performing the index test or the reference standard	n/a
DISCUSSION	26	Study limitations, including sources of potential bias, statistical uncertainty, and generalizability	6
	27	Implications for practice, including the intended use and clinical role of the index test	6,7
OTHER INFORMATION	28	Registration number and name of registry	n/a
	29	Where the full study protocol can be accessed	n/a
	30	Sources of funding and other support; role of funders	7

Section & Topic	No	Item	Page
		**Gaydos 2011[16]**	
TITLE OR ABSTRACT	1	Identification as a study of diagnostic accuracy using at least one measure of accuracy (such as sensitivity, specificity, predictive values, or AUC)	n/a
ABSTRACT	2	Structured summary of study design, methods, results, and conclusions (for specific guidance, see)	1
INTRODUCTION	3	Scientific and clinical background, including the intended use and clinical role of the index test	n/a
	4	Study objectives and hypotheses	2
METHODS Study design	5	Whether data collection was planned before the index test and reference standard were performed (prospective study) or after (retrospective study)	2
Participants	6	Eligibility criteria	2
	7	On what basis potentially eligible participants were identified (such as symptoms, results from previous tests, inclusion in registry)	2
	8	Where and when potentially eligible participants were identified (setting, location and dates)	2
	9	Whether participants formed a consecutive, random or convenience series	2
Test methods	10a STARD for Abstracts	Index test, in sufficient detail to allow replication	2,3
	10b	Reference standard, in sufficient detail to allow replication	n/a
	11	Rationale for choosing the reference standard (if alternatives exist)	n/a
	12a	Definition of and rationale for test positivity cut-offs or result categories of the index test, distinguishing pre-specified from exploratory	n/a
	12b	Definition of and rationale for test positivity cut-offs or result categories of the reference standard, distinguishing pre-specified from exploratory	n/a
	13a	Whether clinical information and reference standard results were available to the performers/readers of the index test	2,3
	13b	Whether clinical information and index test results were available to the assessors of the reference standard	3
Analysis	14	Methods for estimating or comparing measures of diagnostic accuracy	3
	15	How indeterminate index test or reference standard results were handled	n/a
	16	How missing data on the index test and reference standard were handled	n/a
	17	Any analyses of variability in diagnostic accuracy, distinguishing pre-specified from exploratory	n/a
	18	Intended sample size and how it was determined	n/a
RESULTS Participants	19	Flow of participants, using a diagram	n/a
	20	Baseline demographic and clinical characteristics of participants	8
	21a	Distribution of severity of disease in those with the target condition	n/a
	21b	Distribution of alternative diagnoses in those without the target condition	n/a
	22	Time interval and any clinical interventions between index test and reference standard	3
Test results	23	Cross tabulation of the index test results (or their distribution) by the results of the reference standard	n/a
	24	Estimates of diagnostic accuracy and their precision (such as 95% confidence intervals)	n/a
	25	Any adverse events from performing the index test or the reference standard	n/a
DISCUSSION	26	Study limitations, including sources of potential bias, statistical uncertainty, and generalizability	4
	27	Implications for practice, including the intended use and clinical role of the index test	5
OTHER INFORMATION	28	Registration number and name of registry	n/a
	29	Where the full study protocol can be accessed	n/a
	30	Sources of funding and other support; role of funders	5

Section & Topic	No	Item	Page
		**Dong 2014[15]**	
TITLE OR ABSTRACT	1	Identification as a study of diagnostic accuracy using at least one measure of accuracy (such as sensitivity, specificity, predictive values, or AUC)	1
ABSTRACT	2	Structured summary of study design, methods, results, and conclusions (for specific guidance, see)	1
INTRODUCTION	3	Scientific and clinical background, including the intended use and clinical role of the index test	n/a
	4	Study objectives and hypotheses	1
METHODS<br>Study design	5	Whether data collection was planned before the index test and reference standard were performed (prospective study) or after (retrospective study)	n/a
Participants	6	Eligibility criteria	n/a
	7	On what basis potentially eligible participants were identified (such as symptoms, results from previous tests, inclusion in registry)	n/a
	8	Where and when potentially eligible participants were identified (setting, location and dates)	n/a
	9	Whether participants formed a consecutive, random or convenience series	1
Test methods	10a STARD for Abstracts	Index test, in sufficient detail to allow replication	n/a
	10b	Reference standard, in sufficient detail to allow replication	n/a
	11	Rationale for choosing the reference standard (if alternatives exist)	n/a
	12a	Definition of and rationale for test positivity cut-offs or result categories of the index test, distinguishing pre-specified from exploratory	n/a
	12b	Definition of and rationale for test positivity cut-offs or result categories of the reference standard, distinguishing pre-specified from exploratory	n/a
	13a	Whether clinical information and reference standard results were available to the performers/readers of the index test	n/a
	13b	Whether clinical information and index test results were available to the assessors of the reference standard	n/a
Analysis	14	Methods for estimating or comparing measures of diagnostic accuracy	n/a
	15	How indeterminate index test or reference standard results were handled	n/a
	16	How missing data on the index test and reference standard were handled	n/a
	17	Any analyses of variability in diagnostic accuracy, distinguishing pre-specified from exploratory	n/a
	18	Intended sample size and how it was determined	n/a
RESULTS<br>Participants	19	Flow of participants, using a diagram	n/a
	20	Baseline demographic and clinical characteristics of participants	1
	21a	Distribution of severity of disease in those with the target condition	n/a
	21b	Distribution of alternative diagnoses in those without the target condition	n/a
	22	Time interval and any clinical interventions between index test and reference standard	n/a
Test results	23	Cross tabulation of the index test results (or their distribution) by the results of the reference standard	n/a
	24	Estimates of diagnostic accuracy and their precision (such as 95% confidence intervals)	1
	25	Any adverse events from performing the index test or the reference standard	n/a
DISCUSSION	26	Study limitations, including sources of potential bias, statistical uncertainty, and generalizability	n/a
	27	Implications for practice, including the intended use and clinical role of the index test	n/a
OTHER INFORMATION	28	Registration number and name of registry	n/a
	29	Where the full study protocol can be accessed	n/a
	30	Sources of funding and other support; role of funders	1

Section & Topic	No	Item	Page
		**Choko 2015[2]**	
TITLE OR ABSTRACT	1	Identification as a study of diagnostic accuracy using at least one measure of accuracy (such as sensitivity, specificity, predictive values, or AUC)	1
ABSTRACT	2	Structured summary of study design, methods, results, and conclusions (for specific guidance, see)	1,2
INTRODUCTION	3	Scientific and clinical background, including the intended use and clinical role of the index test	2
	4	Study objectives and hypotheses	3
METHODS Study design	5	Whether data collection was planned before the index test and reference standard were performed (prospective study) or after (retrospective study)	3
Participants	6	Eligibility criteria	3
	7	On what basis potentially eligible participants were identified (such as symptoms, results from previous tests, inclusion in registry)	3
	8	Where and when potentially eligible participants were identified (setting, location and dates)	3
	9	Whether participants formed a consecutive, random or convenience series	3
Test methods	10a STARD for Abstracts	Index test, in sufficient detail to allow replication	
	10b	Reference standard, in sufficient detail to allow replication	3,4
	11	Rationale for choosing the reference standard (if alternatives exist)	n/a
	12a	Definition of and rationale for test positivity cut-offs or result categories of the index test, distinguishing pre-specified from exploratory	n/a
	12b	Definition of and rationale for test positivity cut-offs or result categories of the reference standard, distinguishing pre-specified from exploratory	n/a
	13a	Whether clinical information and reference standard results were available to the performers/readers of the index test	4
	13b	Whether clinical information and index test results were available to the assessors of the reference standard	4,5
Analysis	14	Methods for estimating or comparing measures of diagnostic accuracy	5
	15	How indeterminate index test or reference standard results were handled	5
	16	How missing data on the index test and reference standard were handled	5
	17	Any analyses of variability in diagnostic accuracy, distinguishing pre-specified from exploratory	n/a
	18	Intended sample size and how it was determined	n/a
RESULTS Participants	19	Flow of participants, using a diagram	6,7
	20	Baseline demographic and clinical characteristics of participants	8
	21a	Distribution of severity of disease in those with the target condition	n/a
	21b	Distribution of alternative diagnoses in those without the target condition	n/a
	22	Time interval and any clinical interventions between index test and reference standard	n/a
Test results	23	Cross tabulation of the index test results (or their distribution) by the results of the reference standard	13
	24	Estimates of diagnostic accuracy and their precision (such as 95% confidence intervals)	13
	25	Any adverse events from performing the index test or the reference standard	13
DISCUSSION	26	Study limitations, including sources of potential bias, statistical uncertainty, and generalizability	16
	27	Implications for practice, including the intended use and clinical role of the index test	n/a
OTHER INFORMATION	28	Registration number and name of registry	n/a
	29	Where the full study protocol can be accessed	1
	30	Sources of funding and other support; role of funders	1

Section & Topic	No	Item	Page
		**Mavedzenge 2015[23]**	
TITLE OR ABSTRACT	1	Identification as a study of diagnostic accuracy using at least one measure of accuracy (such as sensitivity, specificity, predictive values, or AUC)	n/a
ABSTRACT	2	Structured summary of study design, methods, results, and conclusions (for specific guidance, see)	1
INTRODUCTION	3	Scientific and clinical background, including the intended use and clinical role of the index test	n/a
	4	Study objectives and hypotheses	1
METHODS Study design	5	Whether data collection was planned before the index test and reference standard were performed (prospective study) or after (retrospective study)	1
Participants	6	Eligibility criteria	1
	7	On what basis potentially eligible participants were identified (such as symptoms, results from previous tests, inclusion in registry)	1
	8	Where and when potentially eligible participants were identified (setting, location and dates)	1
	9	Whether participants formed a consecutive, random or convenience series	n/a
Test methods	10a STARD for Abstracts	Index test, in sufficient detail to allow replication	n/a
	10b	Reference standard, in sufficient detail to allow replication	n/a
	11	Rationale for choosing the reference standard (if alternatives exist)	n/a
	12a	Definition of and rationale for test positivity cut-offs or result categories of the index test, distinguishing pre-specified from exploratory	n/a
	12b	Definition of and rationale for test positivity cut-offs or result categories of the reference standard, distinguishing pre-specified from exploratory	n/a
	13a	Whether clinical information and reference standard results were available to the performers/readers of the index test	1
	13b	Whether clinical information and index test results were available to the assessors of the reference standard	1
Analysis	14	Methods for estimating or comparing measures of diagnostic accuracy	n/a
	15	How indeterminate index test or reference standard results were handled	1
	16	How missing data on the index test and reference standard were handled	1
	17	Any analyses of variability in diagnostic accuracy, distinguishing pre-specified from exploratory	n/a
	18	Intended sample size and how it was determined	n/a
RESULTS Participants	19	Flow of participants, using a diagram	n/a
	20	Baseline demographic and clinical characteristics of participants	1
	21a	Distribution of severity of disease in those with the target condition	n/a
	21b	Distribution of alternative diagnoses in those without the target condition	n/a
	22	Time interval and any clinical interventions between index test and reference standard	n/a
Test results	23	Cross tabulation of the index test results (or their distribution) by the results of the reference standard	1
	24	Estimates of diagnostic accuracy and their precision (such as 95% confidence intervals)	1
	25	Any adverse events from performing the index test or the reference standard	n/a
DISCUSSION	26	Study limitations, including sources of potential bias, statistical uncertainty, and generalizability	n/a
	27	Implications for practice, including the intended use and clinical role of the index test	n/a
OTHER INFORMATION	28	Registration number and name of registry	n/a
	29	Where the full study protocol can be accessed	n/a
	30	Sources of funding and other support; role of funders	n/a

Section & Topic	No	Item	Page
		**Prazuck 2016**[7]	
TITLE OR ABSTRACT	1	Identification as a study of diagnostic accuracy using at least one measure of accuracy (such as sensitivity, specificity, predictive values, or AUC)	n/a
ABSTRACT	2	Structured summary of study design, methods, results, and conclusions (for specific guidance, see)	n/a
INTRODUCTION	3	Scientific and clinical background, including the intended use and clinical role of the index test	1,2
	4	Study objectives and hypotheses	2
METHODS<br>Study design	5	Whether data collection was planned before the index test and reference standard were performed (prospective study) or after (retrospective study)	3
Participants	6	Eligibility criteria	4
	7	On what basis potentially eligible participants were identified (such as symptoms, results from previous tests, inclusion in registry)	4
	8	Where and when potentially eligible participants were identified (setting, location and dates)	n/a
	9	Whether participants formed a consecutive, random or convenience series	4
Test methods	10a STARD for Abstracts	Index test, in sufficient detail to allow replication	4
	10b	Reference standard, in sufficient detail to allow replication	n/a
	11	Rationale for choosing the reference standard (if alternatives exist)	n/a
	12a	Definition of and rationale for test positivity cut-offs or result categories of the index test, distinguishing pre-specified from exploratory	5
	12b	Definition of and rationale for test positivity cut-offs or result categories of the reference standard, distinguishing pre-specified from exploratory	n/a
	13a	Whether clinical information and reference standard results were available to the performers/readers of the index test	5
	13b	Whether clinical information and index test results were available to the assessors of the reference standard	5
Analysis	14	Methods for estimating or comparing measures of diagnostic accuracy	n/a
	15	How indeterminate index test or reference standard results were handled	n/a
	16	How missing data on the index test and reference standard were handled	n/a
	17	Any analyses of variability in diagnostic accuracy, distinguishing pre-specified from exploratory	n/a
	18	Intended sample size and how it was determined	n/a
RESULTS<br>Participants	19	Flow of participants, using a diagram	n/a
	20	Baseline demographic and clinical characteristics of participants	19
	21a	Distribution of severity of disease in those with the target condition	n/a
	21b	Distribution of alternative diagnoses in those without the target condition	n/a
	22	Time interval and any clinical interventions between index test and reference standard	n/a
Test results	23	Cross tabulation of the index test results (or their distribution) by the results of the reference standard	n/a
	24	Estimates of diagnostic accuracy and their precision (such as 95% confidence intervals)	n/a
	25	Any adverse events from performing the index test or the reference standard	n/a
DISCUSSION	26	Study limitations, including sources of potential bias, statistical uncertainty, and generalizability	n/a
	27	Implications for practice, including the intended use and clinical role of the index test	11
OTHER INFORMATION	28	Registration number and name of registry	n/a
	29	Where the full study protocol can be accessed	n/a
	30	Sources of funding and other support; role of funders	n/a

Section & Topic	No	Item	Page
		**Nour 2012[21]**	
TITLE OR ABSTRACT	1	Identification as a study of diagnostic accuracy using at least one measure of accuracy (such as sensitivity, specificity, predictive values, or AUC)	1
ABSTRACT	2	Structured summary of study design, methods, results, and conclusions (for specific guidance, see)	1
INTRODUCTION	3	Scientific and clinical background, including the intended use and clinical role of the index test	n/a
	4	Study objectives and hypotheses	2
METHODS<br>Study design	5	Whether data collection was planned before the index test and reference standard were performed (prospective study) or after (retrospective study)	2
Participants	6	Eligibility criteria	2
	7	On what basis potentially eligible participants were identified (such as symptoms, results from previous tests, inclusion in registry)	2
	8	Where and when potentially eligible participants were identified (setting, location and dates)	2
	9	Whether participants formed a consecutive, random or convenience series	2
Test methods	10a STARD for Abstracts	Index test, in sufficient detail to allow replication	2
	10b	Reference standard, in sufficient detail to allow replication	n/a
	11	Rationale for choosing the reference standard (if alternatives exist)	n/a
	12a	Definition of and rationale for test positivity cut-offs or result categories of the index test, distinguishing pre-specified from exploratory	n/a
	12b	Definition of and rationale for test positivity cut-offs or result categories of the reference standard, distinguishing pre-specified from exploratory	n/a
	13a	Whether clinical information and reference standard results were available to the performers/readers of the index test	2,3
	13b	Whether clinical information and index test results were available to the assessors of the reference standard	2,3
Analysis	14	Methods for estimating or comparing measures of diagnostic accuracy	n/a
	15	How indeterminate index test or reference standard results were handled	n/a
	16	How missing data on the index test and reference standard were handled	n/a
	17	Any analyses of variability in diagnostic accuracy, distinguishing pre-specified from exploratory	n/a
	18	Intended sample size and how it was determined	n/a
RESULTS<br>Participants	19	Flow of participants, using a diagram	n/a
	20	Baseline demographic and clinical characteristics of participants	8
	21a	Distribution of severity of disease in those with the target condition	n/a
	21b	Distribution of alternative diagnoses in those without the target condition	n/a
	22	Time interval and any clinical interventions between index test and reference standard	n/a
Test results	23	Cross tabulation of the index test results (or their distribution) by the results of the reference standard	n/a
	24	Estimates of diagnostic accuracy and their precision (such as 95% confidence intervals)	n/a
	25	Any adverse events from performing the index test or the reference standard	n/a
DISCUSSION	26	Study limitations, including sources of potential bias, statistical uncertainty, and generalizability	4
	27	Implications for practice, including the intended use and clinical role of the index test	n/a
OTHER INFORMATION	28	Registration number and name of registry	n/a
	29	Where the full study protocol can be accessed	n/a
	30	Sources of funding and other support; role of funders	4

Section & Topic	No	Item	Page
		**Pant Pai 2013[9]**	
TITLE OR ABSTRACT	1	Identification as a study of diagnostic accuracy using at least one measure of accuracy (such as sensitivity, specificity, predictive values, or AUC)	n/a
ABSTRACT	2	Structured summary of study design, methods, results, and conclusions (for specific guidance, see)	1
INTRODUCTION	3	Scientific and clinical background, including the intended use and clinical role of the index test	3
	4	Study objectives and hypotheses	3
METHODS Study design	5	Whether data collection was planned before the index test and reference standard were performed (prospective study) or after (retrospective study)	3
Participants	6	Eligibility criteria	3
	7	On what basis potentially eligible participants were identified (such as symptoms, results from previous tests, inclusion in registry)	3
	8	Where and when potentially eligible participants were identified (setting, location and dates)	3
	9	Whether participants formed a consecutive, random or convenience series	3
Test methods	10a STARD for Abstracts	Index test, in sufficient detail to allow replication	3,4
	10b	Reference standard, in sufficient detail to allow replication	5
	11	Rationale for choosing the reference standard (if alternatives exist)	n/a
	12a	Definition of and rationale for test positivity cut-offs or result categories of the index test, distinguishing pre-specified from exploratory	5
	12b	Definition of and rationale for test positivity cut-offs or result categories of the reference standard, distinguishing pre-specified from exploratory	n/a
	13a	Whether clinical information and reference standard results were available to the performers/readers of the index test	5
	13b	Whether clinical information and index test results were available to the assessors of the reference standard	5
Analysis	14	Methods for estimating or comparing measures of diagnostic accuracy	n/a
	15	How indeterminate index test or reference standard results were handled	n/a
	16	How missing data on the index test and reference standard were handled	n/a
	17	Any analyses of variability in diagnostic accuracy, distinguishing pre-specified from exploratory	n/a
	18	Intended sample size and how it was determined	n/a
RESULTS Participants	19	Flow of participants, using a diagram	4
	20	Baseline demographic and clinical characteristics of participants	5,6
	21a	Distribution of severity of disease in those with the target condition	n/a
	21b	Distribution of alternative diagnoses in those without the target condition	n/a
	22	Time interval and any clinical interventions between index test and reference standard	n/a
Test results	23	Cross tabulation of the index test results (or their distribution) by the results of the reference standard	n/a
	24	Estimates of diagnostic accuracy and their precision (such as 95% confidence intervals)	5
	25	Any adverse events from performing the index test or the reference standard	n/a
DISCUSSION	26	Study limitations, including sources of potential bias, statistical uncertainty, and generalizability	8
	27	Implications for practice, including the intended use and clinical role of the index test	n/a
OTHER INFORMATION	28	Registration number and name of registry	n/a
	29	Where the full study protocol can be accessed	n/a
	30	Sources of funding and other support; role of funders	1

Section & Topic	No	Item	Page
		**Choko 2011[3]**	
TITLE OR ABSTRACT	1	Identification as a study of diagnostic accuracy using at least one measure of accuracy (such as sensitivity, specificity, predictive values, or AUC)	1
ABSTRACT	2	Structured summary of study design, methods, results, and conclusions (for specific guidance, see)	1
INTRODUCTION	3	Scientific and clinical background, including the intended use and clinical role of the index test	2
	4	Study objectives and hypotheses	2
METHODS<br>Study design	5	Whether data collection was planned before the index test and reference standard were performed (prospective study) or after (retrospective study)	2
Participants	6	Eligibility criteria	2
	7	On what basis potentially eligible participants were identified (such as symptoms, results from previous tests, inclusion in registry)	2
	8	Where and when potentially eligible participants were identified (setting, location and dates)	2
	9	Whether participants formed a consecutive, random or convenience series	2
Test methods	10a STARD for Abstracts	Index test, in sufficient detail to allow replication	2,3
	10b	Reference standard, in sufficient detail to allow replication	
	11	Rationale for choosing the reference standard (if alternatives exist)	n/a
	12a	Definition of and rationale for test positivity cut-offs or result categories of the index test, distinguishing pre-specified from exploratory	4
	12b	Definition of and rationale for test positivity cut-offs or result categories of the reference standard, distinguishing pre-specified from exploratory	n/a
	13a	Whether clinical information and reference standard results were available to the performers/readers of the index test	4
	13b	Whether clinical information and index test results were available to the assessors of the reference standard	4
Analysis	14	Methods for estimating or comparing measures of diagnostic accuracy	4
	15	How indeterminate index test or reference standard results were handled	4
	16	How missing data on the index test and reference standard were handled	4
	17	Any analyses of variability in diagnostic accuracy, distinguishing pre-specified from exploratory	n/a
	18	Intended sample size and how it was determined	4
RESULTS<br>Participants	19	Flow of participants, using a diagram	6
	20	Baseline demographic and clinical characteristics of participants	5
	21a	Distribution of severity of disease in those with the target condition	n/a
	21b	Distribution of alternative diagnoses in those without the target condition	n/a
	22	Time interval and any clinical interventions between index test and reference standard	n/a
Test results	23	Cross tabulation of the index test results (or their distribution) by the results of the reference standard	6
	24	Estimates of diagnostic accuracy and their precision (such as 95% confidence intervals)	5
	25	Any adverse events from performing the index test or the reference standard	n/a
DISCUSSION	26	Study limitations, including sources of potential bias, statistical uncertainty, and generalizability	9
	27	Implications for practice, including the intended use and clinical role of the index test	9
OTHER INFORMATION	28	Registration number and name of registry	n/a
	29	Where the full study protocol can be accessed	n/a
	30	Sources of funding and other support; role of funders	1

Section & Topic	No	Item	Page
		**Martínez-Perez 2016[6]**	
TITLE OR ABSTRACT	1	Identification as a study of diagnostic accuracy using at least one measure of accuracy (such as sensitivity, specificity, predictive values, or AUC)	1
ABSTRACT	2	Structured summary of study design, methods, results, and conclusions (for specific guidance, see)	1
INTRODUCTION	3	Scientific and clinical background, including the intended use and clinical role of the index test	n/a
	4	Study objectives and hypotheses	1
METHODS Study design	5	Whether data collection was planned before the index test and reference standard were performed (prospective study) or after (retrospective study)	1
Participants	6	Eligibility criteria	1
	7	On what basis potentially eligible participants were identified (such as symptoms, results from previous tests, inclusion in registry)	1
	8	Where and when potentially eligible participants were identified (setting, location and dates)	1
	9	Whether participants formed a consecutive, random or convenience series	1
Test methods	10a STARD for Abstracts	Index test, in sufficient detail to allow replication	1
	10b	Reference standard, in sufficient detail to allow replication	1
	11	Rationale for choosing the reference standard (if alternatives exist)	n/a
	12a	Definition of and rationale for test positivity cut-offs or result categories of the index test, distinguishing pre-specified from exploratory	n/a
	12b	Definition of and rationale for test positivity cut-offs or result categories of the reference standard, distinguishing pre-specified from exploratory	n/a
	13a	Whether clinical information and reference standard results were available to the performers/readers of the index test	1
	13b	Whether clinical information and index test results were available to the assessors of the reference standard	1
Analysis	14	Methods for estimating or comparing measures of diagnostic accuracy	5
	15	How indeterminate index test or reference standard results were handled	4
	16	How missing data on the index test and reference standard were handled	n/a
	17	Any analyses of variability in diagnostic accuracy, distinguishing pre-specified from exploratory	n/a
	18	Intended sample size and how it was determined	4
RESULTS Participants	19	Flow of participants, using a diagram	17
	20	Baseline demographic and clinical characteristics of participants	5-6
	21a	Distribution of severity of disease in those with the target condition	n/a
	21b	Distribution of alternative diagnoses in those without the target condition	n/a
	22	Time interval and any clinical interventions between index test and reference standard	n/a
Test results	23	Cross tabulation of the index test results (or their distribution) by the results of the reference standard	14-15
	24	Estimates of diagnostic accuracy and their precision (such as 95% confidence intervals)	6, 15
	25	Any adverse events from performing the index test or the reference standard	n/a
DISCUSSION	26	Study limitations, including sources of potential bias, statistical uncertainty, and generalizability	8-9
	27	Implications for practice, including the intended use and clinical role of the index test	9
OTHER INFORMATION	28	Registration number and name of registry	n/a
	29	Where the full study protocol can be accessed	n/a
	30	Sources of funding and other support; role of funders	n/a

Section & Topic	No	Item	Page
		**Sarkar 2016[8]**	
TITLE OR ABSTRACT	1	Identification as a study of diagnostic accuracy using at least one measure of accuracy (such as sensitivity, specificity, predictive values, or AUC)	n/a
ABSTRACT	2	Structured summary of study design, methods, results, and conclusions (for specific guidance, see)	1
INTRODUCTION	3	Scientific and clinical background, including the intended use and clinical role of the index test	n/a
	4	Study objectives and hypotheses	1
METHODS Study design	5	Whether data collection was planned before the index test and reference standard were performed (prospective study) or after (retrospective study)	1
Participants	6	Eligibility criteria	1
	7	On what basis potentially eligible participants were identified (such as symptoms, results from previous tests, inclusion in registry)	1
	8	Where and when potentially eligible participants were identified (setting, location and dates)	n/a
	9	Whether participants formed a consecutive, random or convenience series	1
Test methods	10a STARD for Abstracts	Index test, in sufficient detail to allow replication	n/a
	10b	Reference standard, in sufficient detail to allow replication	n/a
	11	Rationale for choosing the reference standard (if alternatives exist)	n/a
	12a	Definition of and rationale for test positivity cut-offs or result categories of the index test, distinguishing pre-specified from exploratory	n/a
	12b	Definition of and rationale for test positivity cut-offs or result categories of the reference standard, distinguishing pre-specified from exploratory	n/a
	13a	Whether clinical information and reference standard results were available to the performers/readers of the index test	1
	13b	Whether clinical information and index test results were available to the assessors of the reference standard	1
Analysis	14	Methods for estimating or comparing measures of diagnostic accuracy	1
	15	How indeterminate index test or reference standard results were handled	n/a
	16	How missing data on the index test and reference standard were handled	n/a
	17	Any analyses of variability in diagnostic accuracy, distinguishing pre-specified from exploratory	n/a
	18	Intended sample size and how it was determined	n/a
RESULTS Participants	19	Flow of participants, using a diagram	1
	20	Baseline demographic and clinical characteristics of participants	n/a
	21a	Distribution of severity of disease in those with the target condition	n/a
	21b	Distribution of alternative diagnoses in those without the target condition	n/a
	22	Time interval and any clinical interventions between index test and reference standard	n/a
Test results	23	Cross tabulation of the index test results (or their distribution) by the results of the reference standard	1
	24	Estimates of diagnostic accuracy and their precision (such as 95% confidence intervals)	1
	25	Any adverse events from performing the index test or the reference standard	n/a
DISCUSSION	26	Study limitations, including sources of potential bias, statistical uncertainty, and generalizability	n/a
	27	Implications for practice, including the intended use and clinical role of the index test	n/a
OTHER INFORMATION	28	Registration number and name of registry	n/a
	29	Where the full study protocol can be accessed	n/a
	30	Sources of funding and other support; role of funders	n/a

Section & Topic	No	Item	Page
		**FDA phase 2b 2012 observed arm**[25]	
TITLE OR ABSTRACT	1	Identification as a study of diagnostic accuracy using at least one measure of accuracy (such as sensitivity, specificity, predictive values, or AUC)	n/a
ABSTRACT	2	Structured summary of study design, methods, results, and conclusions (for specific guidance, see)	n/a
INTRODUCTION	3	Scientific and clinical background, including the intended use and clinical role of the index test	3-5
	4	Study objectives and hypotheses	14-15
METHODS<br>Study design	5	Whether data collection was planned before the index test and reference standard were performed (prospective study) or after (retrospective study)	16
Participants	6	Eligibility criteria	16
	7	On what basis potentially eligible participants were identified (such as symptoms, results from previous tests, inclusion in registry)	16
	8	Where and when potentially eligible participants were identified (setting, location and dates)	n/a
	9	Whether participants formed a consecutive, random or convenience series	18
Test methods	10a<br>STARD for Abstracts	Index test, in sufficient detail to allow replication	16-17
	10b	Reference standard, in sufficient detail to allow replication	19-20
	11	Rationale for choosing the reference standard (if alternatives exist)	19
	12a	Definition of and rationale for test positivity cut-offs or result categories of the index test, distinguishing pre-specified from exploratory	22
	12b	Definition of and rationale for test positivity cut-offs or result categories of the reference standard, distinguishing pre-specified from exploratory	19
	13a	Whether clinical information and reference standard results were available to the performers/readers of the index test	19-20
	13b	Whether clinical information and index test results were available to the assessors of the reference standard	19-20
Analysis	14	Methods for estimating or comparing measures of diagnostic accuracy	20
	15	How indeterminate index test or reference standard results were handled	22
	16	How missing data on the index test and reference standard were handled	22
	17	Any analyses of variability in diagnostic accuracy, distinguishing pre-specified from exploratory	n/a
	18	Intended sample size and how it was determined	n/a
RESULTS<br>Participants	19	Flow of participants, using a diagram	n/a
	20	Baseline demographic and clinical characteristics of participants	21
	21a	Distribution of severity of disease in those with the target condition	n/a
	21b	Distribution of alternative diagnoses in those without the target condition	n/a
	22	Time interval and any clinical interventions between index test and reference standard	n/a
Test results	23	Cross tabulation of the index test results (or their distribution) by the results of the reference standard	23
	24	Estimates of diagnostic accuracy and their precision (such as 95% confidence intervals)	22-23
	25	Any adverse events from performing the index test or the reference standard	n/a
DISCUSSION	26	Study limitations, including sources of potential bias, statistical uncertainty, and generalizability	25
	27	Implications for practice, including the intended use and clinical role of the index test	25
OTHER INFORMATION	28	Registration number and name of registry	1
	29	Where the full study protocol can be accessed	n/a
	30	Sources of funding and other support; role of funders	n/a

Section & Topic	No	Item	Page
		**FDA phase 3 2012 unobserved arm[25]**	
TITLE OR ABSTRACT	1	Identification as a study of diagnostic accuracy using at least one measure of accuracy (such as sensitivity, specificity, predictive values, or AUC)	n/a
ABSTRACT	2	Structured summary of study design, methods, results, and conclusions (for specific guidance, see)	n/a
INTRODUCTION	3	Scientific and clinical background, including the intended use and clinical role of the index test	3-5
	4	Study objectives and hypotheses	15
METHODS Study design	5	Whether data collection was planned before the index test and reference standard were performed (prospective study) or after (retrospective study)	15
Participants	6	Eligibility criteria	25-26
	7	On what basis potentially eligible participants were identified (such as symptoms, results from previous tests, inclusion in registry)	26
	8	Where and when potentially eligible participants were identified (setting, location and dates)	n/a
	9	Whether participants formed a consecutive, random or convenience series	26
Test methods	10a STARD for Abstracts	Index test, in sufficient detail to allow replication	26
	10b	Reference standard, in sufficient detail to allow replication	32
	11	Rationale for choosing the reference standard (if alternatives exist)	n/a
	12a	Definition of and rationale for test positivity cut-offs or result categories of the index test, distinguishing pre-specified from exploratory	n/a
	12b	Definition of and rationale for test positivity cut-offs or result categories of the reference standard, distinguishing pre-specified from exploratory	33
	13a	Whether clinical information and reference standard results were available to the performers/readers of the index test	n/a
	13b	Whether clinical information and index test results were available to the assessors of the reference standard	n/a
Analysis	14	Methods for estimating or comparing measures of diagnostic accuracy	26
	15	How indeterminate index test or reference standard results were handled	26
	16	How missing data on the index test and reference standard were handled	31-32
	17	Any analyses of variability in diagnostic accuracy, distinguishing pre-specified from exploratory	n/a
	18	Intended sample size and how it was determined	n/a
RESULTS Participants	19	Flow of participants, using a diagram	31
	20	Baseline demographic and clinical characteristics of participants	28
	21a	Distribution of severity of disease in those with the target condition	n/a
	21b	Distribution of alternative diagnoses in those without the target condition	n/a
	22	Time interval and any clinical interventions between index test and reference standard	n/a
Test results	23	Cross tabulation of the index test results (or their distribution) by the results of the reference standard	35
	24	Estimates of diagnostic accuracy and their precision (such as 95% confidence intervals)	36
	25	Any adverse events from performing the index test or the reference standard	37-38
DISCUSSION	26	Study limitations, including sources of potential bias, statistical uncertainty, and generalizability	n/a
	27	Implications for practice, including the intended use and clinical role of the index test	41
OTHER INFORMATION	28	Registration number and name of registry	1
	29	Where the full study protocol can be accessed	n/a
	30	Sources of funding and other support; role of funders	n/a

Section & Topic	No	Item	Page
		**Majam**[4]	
TITLE OR ABSTRACT	1	Identification as a study of diagnostic accuracy using at least one measure of accuracy (such as sensitivity, specificity, predictive values, or AUC)	n/a
ABSTRACT	2	Structured summary of study design, methods, results, and conclusions (for specific guidance, see)	1
INTRODUCTION	3	Scientific and clinical background, including the intended use and clinical role of the index test	n/a
	4	Study objectives and hypotheses	
METHODS Study design	5	Whether data collection was planned before the index test and reference standard were performed (prospective study) or after (retrospective study)	1
Participants	6	Eligibility criteria	1
	7	On what basis potentially eligible participants were identified (such as symptoms, results from previous tests, inclusion in registry)	1
	8	Where and when potentially eligible participants were identified (setting, location and dates)	n/a
	9	Whether participants formed a consecutive, random or convenience series	1
Test methods	10a STARD for Abstracts	Index test, in sufficient detail to allow replication	
	10b	Reference standard, in sufficient detail to allow replication	n/a
	11	Rationale for choosing the reference standard (if alternatives exist)	n/a
	12a	Definition of and rationale for test positivity cut-offs or result categories of the index test, distinguishing pre-specified from exploratory	n/a
	12b	Definition of and rationale for test positivity cut-offs or result categories of the reference standard, distinguishing pre-specified from exploratory	n/a
	13a	Whether clinical information and reference standard results were available to the performers/readers of the index test	n/a
	13b	Whether clinical information and index test results were available to the assessors of the reference standard	n/a
Analysis	14	Methods for estimating or comparing measures of diagnostic accuracy	n/a
	15	How indeterminate index test or reference standard results were handled	n/a
	16	How missing data on the index test and reference standard were handled	n/a
	17	Any analyses of variability in diagnostic accuracy, distinguishing pre-specified from exploratory	n/a
	18	Intended sample size and how it was determined	n/a
RESULTS Participants	19	Flow of participants, using a diagram	n/a
	20	Baseline demographic and clinical characteristics of participants	1-2
	21a	Distribution of severity of disease in those with the target condition	n/a
	21b	Distribution of alternative diagnoses in those without the target condition	n/a
	22	Time interval and any clinical interventions between index test and reference standard	n/a
Test results	23	Cross tabulation of the index test results (or their distribution) by the results of the reference standard	n/a
	24	Estimates of diagnostic accuracy and their precision (such as 95% confidence intervals)	n/a
	25	Any adverse events from performing the index test or the reference standard	n/a
DISCUSSION	26	Study limitations, including sources of potential bias, statistical uncertainty, and generalizability	n/a
	27	Implications for practice, including the intended use and clinical role of the index test	n/a
OTHER INFORMATION	28	Registration number and name of registry	n/a
	29	Where the full study protocol can be accessed	n/a
	30	Sources of funding and other support; role of funders	n/a

Section & Topic	No	Item	Page
		**Gaydos 2013[17]**	
TITLE OR ABSTRACT	1	Identification as a study of diagnostic accuracy using at least one measure of accuracy (such as sensitivity, specificity, predictive values, or AUC)	n/a
ABSTRACT	2	Structured summary of study design, methods, results, and conclusions (for specific guidance, see)	1
INTRODUCTION	3	Scientific and clinical background, including the intended use and clinical role of the index test	n/a
	4	Study objectives and hypotheses	2
METHODS Study design	5	Whether data collection was planned before the index test and reference standard were performed (prospective study) or after (retrospective study)	2
Participants	6	Eligibility criteria	2-3
	7	On what basis potentially eligible participants were identified (such as symptoms, results from previous tests, inclusion in registry)	2-3
	8	Where and when potentially eligible participants were identified (setting, location and dates)	2-3
	9	Whether participants formed a consecutive, random or convenience series	3
Test methods	10a STARD for Abstracts	Index test, in sufficient detail to allow replication	3
	10b	Reference standard, in sufficient detail to allow replication	3
	11	Rationale for choosing the reference standard (if alternatives exist)	n/a
	12a	Definition of and rationale for test positivity cut-offs or result categories of the index test, distinguishing pre-specified from exploratory	n/a
	12b	Definition of and rationale for test positivity cut-offs or result categories of the reference standard, distinguishing pre-specified from exploratory	n/a
	13a	Whether clinical information and reference standard results were available to the performers/readers of the index test	3
	13b	Whether clinical information and index test results were available to the assessors of the reference standard	3
Analysis	14	Methods for estimating or comparing measures of diagnostic accuracy	n/a
	15	How indeterminate index test or reference standard results were handled	n/a
	16	How missing data on the index test and reference standard were handled	n/a
	17	Any analyses of variability in diagnostic accuracy, distinguishing pre-specified from exploratory	n/a
	18	Intended sample size and how it was determined	n/a
RESULTS Participants	19	Flow of participants, using a diagram	n/a
	20	Baseline demographic and clinical characteristics of participants	n/a
	21a	Distribution of severity of disease in those with the target condition	n/a
	21b	Distribution of alternative diagnoses in those without the target condition	n/a
	22	Time interval and any clinical interventions between index test and reference standard	3
Test results	23	Cross tabulation of the index test results (or their distribution) by the results of the reference standard	n/a
	24	Estimates of diagnostic accuracy and their precision (such as 95% confidence intervals)	n/a
	25	Any adverse events from performing the index test or the reference standard	n/a
DISCUSSION	26	Study limitations, including sources of potential bias, statistical uncertainty, and generalizability	5
	27	Implications for practice, including the intended use and clinical role of the index test	n/a
OTHER INFORMATION	28	Registration number and name of registry	n/a
	29	Where the full study protocol can be accessed	n/a
	30	Sources of funding and other support; role of funders	5

Section & Topic	No	Item	Page
		**Spielberg 2003[22]**	
TITLE OR ABSTRACT	1	Identification as a study of diagnostic accuracy using at least one measure of accuracy (such as sensitivity, specificity, predictive values, or AUC)	n/a
ABSTRACT	2	Structured summary of study design, methods, results, and conclusions (for specific guidance, see)	1
INTRODUCTION	3	Scientific and clinical background, including the intended use and clinical role of the index test	n/a
	4	Study objectives and hypotheses	1
METHODS Study design	5	Whether data collection was planned before the index test and reference standard were performed (prospective study) or after (retrospective study)	1
Participants	6	Eligibility criteria	n/a
	7	On what basis potentially eligible participants were identified (such as symptoms, results from previous tests, inclusion in registry)	n/a
	8	Where and when potentially eligible participants were identified (setting, location and dates)	n/a
	9	Whether participants formed a consecutive, random or convenience series	1
Test methods	10a STARD for Abstracts	Index test, in sufficient detail to allow replication	n/a
	10b	Reference standard, in sufficient detail to allow replication	n/a
	11	Rationale for choosing the reference standard (if alternatives exist)	n/a
	12a	Definition of and rationale for test positivity cut-offs or result categories of the index test, distinguishing pre-specified from exploratory	n/a
	12b	Definition of and rationale for test positivity cut-offs or result categories of the reference standard, distinguishing pre-specified from exploratory	n/a
	13a	Whether clinical information and reference standard results were available to the performers/readers of the index test	1
	13b	Whether clinical information and index test results were available to the assessors of the reference standard	1
Analysis	14	Methods for estimating or comparing measures of diagnostic accuracy	n/a
	15	How indeterminate index test or reference standard results were handled	n/a
	16	How missing data on the index test and reference standard were handled	n/a
	17	Any analyses of variability in diagnostic accuracy, distinguishing pre-specified from exploratory	n/a
	18	Intended sample size and how it was determined	n/a
RESULTS Participants	19	Flow of participants, using a diagram	n/a
	20	Baseline demographic and clinical characteristics of participants	n/a
	21a	Distribution of severity of disease in those with the target condition	n/a
	21b	Distribution of alternative diagnoses in those without the target condition	n/a
	22	Time interval and any clinical interventions between index test and reference standard	n/a
Test results	23	Cross tabulation of the index test results (or their distribution) by the results of the reference standard	n/a
	24	Estimates of diagnostic accuracy and their precision (such as 95% confidence intervals)	n/a
	25	Any adverse events from performing the index test or the reference standard	n/a
DISCUSSION	26	Study limitations, including sources of potential bias, statistical uncertainty, and generalizability	n/a
	27	Implications for practice, including the intended use and clinical role of the index test	n/a
OTHER INFORMATION	28	Registration number and name of registry	n/a
	29	Where the full study protocol can be accessed	n/a
	30	Sources of funding and other support; role of funders	n/a

Section & Topic	No	Item	Page
		**Chavez 2016[14]**	
TITLE OR ABSTRACT	1	Identification as a study of diagnostic accuracy using at least one measure of accuracy (such as sensitivity, specificity, predictive values, or AUC)	1
ABSTRACT	2	Structured summary of study design, methods, results, and conclusions (for specific guidance, see)	2-5
INTRODUCTION	3	Scientific and clinical background, including the intended use and clinical role of the index test	n/a
	4	Study objectives and hypotheses	4
METHODS Study design	5	Whether data collection was planned before the index test and reference standard were performed (prospective study) or after (retrospective study)	3
Participants	6	Eligibility criteria	8
	7	On what basis potentially eligible participants were identified (such as symptoms, results from previous tests, inclusion in registry)	n/a
	8	Where and when potentially eligible participants were identified (setting, location and dates)	2
	9	Whether participants formed a consecutive, random or convenience series	2
Test methods	10a STARD for Abstracts	Index test, in sufficient detail to allow replication	10-12
	10b	Reference standard, in sufficient detail to allow replication	10-12
	11	Rationale for choosing the reference standard (if alternatives exist)	n/a
	12a	Definition of and rationale for test positivity cut-offs or result categories of the index test, distinguishing pre-specified from exploratory	n/a
	12b	Definition of and rationale for test positivity cut-offs or result categories of the reference standard, distinguishing pre-specified from exploratory	n/a
	13a	Whether clinical information and reference standard results were available to the performers/readers of the index test	n/a
	13b	Whether clinical information and index test results were available to the assessors of the reference standard	n/a
Analysis	14	Methods for estimating or comparing measures of diagnostic accuracy	n/a
	15	How indeterminate index test or reference standard results were handled	n/a
	16	How missing data on the index test and reference standard were handled	n/a
	17	Any analyses of variability in diagnostic accuracy, distinguishing pre-specified from exploratory	n/a
	18	Intended sample size and how it was determined	n/a
RESULTS Participants	19	Flow of participants, using a diagram	n/a
	20	Baseline demographic and clinical characteristics of participants	15
	21a	Distribution of severity of disease in those with the target condition	n/a
	21b	Distribution of alternative diagnoses in those without the target condition	n/a
	22	Time interval and any clinical interventions between index test and reference standard	n/a
Test results	23	Cross tabulation of the index test results (or their distribution) by the results of the reference standard	25-26
	24	Estimates of diagnostic accuracy and their precision (such as 95% confidence intervals)	24
	25	Any adverse events from performing the index test or the reference standard	n/a
DISCUSSION	26	Study limitations, including sources of potential bias, statistical uncertainty, and generalizability	31
	27	Implications for practice, including the intended use and clinical role of the index test	n/a
OTHER INFORMATION	28	Registration number and name of registry	n/a
	29	Where the full study protocol can be accessed	n/a
	30	Sources of funding and other support; role of funders	n/a

Section & Topic	No	Item	Page
		**MacGowan 2014**[1]	
TITLE OR ABSTRACT	1	Identification as a study of diagnostic accuracy using at least one measure of accuracy (such as sensitivity, specificity, predictive values, or AUC)	n/a
ABSTRACT	2	Structured summary of study design, methods, results, and conclusions (for specific guidance, see)	1
INTRODUCTION	3	Scientific and clinical background, including the intended use and clinical role of the index test	1
	4	Study objectives and hypotheses	1
METHODS Study design	5	Whether data collection was planned before the index test and reference standard were performed (prospective study) or after (retrospective study)	1
Participants	6	Eligibility criteria	n/a
	7	On what basis potentially eligible participants were identified (such as symptoms, results from previous tests, inclusion in registry)	n/a
	8	Where and when potentially eligible participants were identified (setting, location and dates)	1
	9	Whether participants formed a consecutive, random or convenience series	1
Test methods	10a STARD for Abstracts	Index test, in sufficient detail to allow replication	1
	10b	Reference standard, in sufficient detail to allow replication	1
	11	Rationale for choosing the reference standard (if alternatives exist)	n/a
	12a	Definition of and rationale for test positivity cut-offs or result categories of the index test, distinguishing pre-specified from exploratory	n/a
	12b	Definition of and rationale for test positivity cut-offs or result categories of the reference standard, distinguishing pre-specified from exploratory	n/a
	13a	Whether clinical information and reference standard results were available to the performers/readers of the index test	1
	13b	Whether clinical information and index test results were available to the assessors of the reference standard	1
Analysis	14	Methods for estimating or comparing measures of diagnostic accuracy	n/a
	15	How indeterminate index test or reference standard results were handled	n/a
	16	How missing data on the index test and reference standard were handled	1
	17	Any analyses of variability in diagnostic accuracy, distinguishing pre-specified from exploratory	n/a
	18	Intended sample size and how it was determined	n/a
RESULTS Participants	19	Flow of participants, using a diagram	n/a
	20	Baseline demographic and clinical characteristics of participants	1
	21a	Distribution of severity of disease in those with the target condition	n/a
	21b	Distribution of alternative diagnoses in those without the target condition	n/a
	22	Time interval and any clinical interventions between index test and reference standard	n/a
Test results	23	Cross tabulation of the index test results (or their distribution) by the results of the reference standard	1
	24	Estimates of diagnostic accuracy and their precision (such as 95% confidence intervals)	n/a
	25	Any adverse events from performing the index test or the reference standard	n/a
DISCUSSION	26	Study limitations, including sources of potential bias, statistical uncertainty, and generalizability	1
	27	Implications for practice, including the intended use and clinical role of the index test	n/a
OTHER INFORMATION	28	Registration number and name of registry	n/a
	29	Where the full study protocol can be accessed	n/a
	30	Sources of funding and other support; role of funders	1

Section & Topic	No	Item	Page
		**Li 2016**[20]	
TITLE OR ABSTRACT	1	Identification as a study of diagnostic accuracy using at least one measure of accuracy (such as sensitivity, specificity, predictive values, or AUC)	1
ABSTRACT	2	Structured summary of study design, methods, results, and conclusions (for specific guidance, see)	1
INTRODUCTION	3	Scientific and clinical background, including the intended use and clinical role of the index test	1
	4	Study objectives and hypotheses	1
METHODS Study design	5	Whether data collection was planned before the index test and reference standard were performed (prospective study) or after (retrospective study)	1
Participants	6	Eligibility criteria	1
	7	On what basis potentially eligible participants were identified (such as symptoms, results from previous tests, inclusion in registry)	n/a
	8	Where and when potentially eligible participants were identified (setting, location and dates)	1,2
	9	Whether participants formed a consecutive, random or convenience series	2
Test methods	10a STARD for Abstracts	Index test, in sufficient detail to allow replication	2
	10b	Reference standard, in sufficient detail to allow replication	2
	11	Rationale for choosing the reference standard (if alternatives exist)	n/a
	12a	Definition of and rationale for test positivity cut-offs or result categories of the index test, distinguishing pre-specified from exploratory	n/a
	12b	Definition of and rationale for test positivity cut-offs or result categories of the reference standard, distinguishing pre-specified from exploratory	n/a
	13a	Whether clinical information and reference standard results were available to the performers/readers of the index test	n/a
	13b	Whether clinical information and index test results were available to the assessors of the reference standard	2
Analysis	14	Methods for estimating or comparing measures of diagnostic accuracy	n/a
	15	How indeterminate index test or reference standard results were handled	n/a
	16	How missing data on the index test and reference standard were handled	n/a
	17	Any analyses of variability in diagnostic accuracy, distinguishing pre-specified from exploratory	n/a
	18	Intended sample size and how it was determined	n/a
RESULTS Participants	19	Flow of participants, using a diagram	n/a
	20	Baseline demographic and clinical characteristics of participants	2
	21a	Distribution of severity of disease in those with the target condition	n/a
	21b	Distribution of alternative diagnoses in those without the target condition	n/a
	22	Time interval and any clinical interventions between index test and reference standard	n/a
Test results	23	Cross tabulation of the index test results (or their distribution) by the results of the reference standard	3
	24	Estimates of diagnostic accuracy and their precision (such as 95% confidence intervals)	3
	25	Any adverse events from performing the index test or the reference standard	n/a
DISCUSSION	26	Study limitations, including sources of potential bias, statistical uncertainty, and generalizability	5
	27	Implications for practice, including the intended use and clinical role of the index test	5
OTHER INFORMATION	28	Registration number and name of registry	n/a
	29	Where the full study protocol can be accessed	n/a
	30	Sources of funding and other support; role of funders	n/a

References

1. MacGowan R, Chavez P, Freeman A, et al. Pilot evaluation of men who have sex with men's ability to self-administer rapid HIV tests and interpret test results. *American Public Health Association Conference*; Atlanta, GA, USA; Nov 15–19, 2014. 298247.
2. Choko AT, MacPherson P, Webb EL, et al. Uptake, accuracy, safety, and linkage into care over two years of promoting annual self-testing for HIV in Blantyre, Malawi: a community-based prospective study. *PLoS Med* 2015; **12**: e1001873.
3. Choko AT, Desmond N, Webb EL, et al. The uptake and accuracy of oral kits for HIV self-testing in high HIV prevalence setting: a cross-sectional feasibility study in Blantyre, Malawi. *PLoS Med* 2011; **8**: 10 e1001102.
4. Majam M. HIV self-Testing in South Africa: the current landscape. Treatment optimization continuing medical education meeting; Durban, South Africa; Apr 8, 2017.
http://www.sahivsoc.org/Files/HIV%20SelfTesting%20in%20SA%20Presentation%20to%20SAHIVSOC_8%20Apr%202017.pdf
5. Marley G, Kang D, Wilson EC, et al. Introducing rapid oral-fluid HIV testing among high risk populations in Shandong, China: feasibility and challenges. *BMC Public Health* 2014; **14**: 422.
6. Martínez-Pérez G, Steele SJ, Govender I, et al. Supervised oral HIV self-testing is accurate in rural KwaZulu Natal, South Africa. *Trop Med Inter Health* 2016; **21**: 759–67.
7. Prazuck T, Karon S, Gubavu C, et al. A finger-stick whole-blood HIV self-test as an HIV screening tool adapted to the general public. *PLoS One* 2016; **11**: e0146755.
8. Sarkar A, Mburu G, Shivkumar PV, et al. Feasibility of supervised self-testing using an oral fluid-based HIV rapid testing method: a cross-sectional, mixed method study among pregnant women in rural India. *J Int AIDS Soc* 2016; **19**: 20993.
9. Pant Pai N, Behlim T, Abrahams L, et al. Will an unsupervised self-testing strategy for HIV work in health care workers of South Africa? A cross sectional pilot feasibility study. *PLoS One* 2013; **8**: e79772.
10. Pant Pai N, Bhargava M, Joseph L, et al. Will an unsupervised self-testing strategy be feasible to operationalize in Canada? Results from a pilot study in students of a large Canadian university. *AIDS Res Treat* 2014; **2014**: 747619
11. Asiimwe S, Oloya J, Song X, Whalen CC. Accuracy of un-supervised versus provider-supervised self-administered HIV testing in Uganda: a randomized implementation trial. *AIDS Behav* 2014; **18**: 2477–84.
12. de la Fuente L, Rosales-Statkus ME, Hoyos J, et al. Are participants in a street-based HIV testing program able to perform their own rapid test and interpret the results? *PLoS One* 2012; **7**: e46555.
13. Lee VJ, Tan SC, Earnest A, Seong PS, Tan HH, Leo YS. User acceptability and feasibility of self-testing with HIV rapid tests. *J Acquir Immune Defic Syndr* 2007; **45**: 449–53.
14. Chavez P, Wesolowski L, Owen M, Gravens L, Sullivan P, MacGowan R. Perceptions and performance of self-administered rapid HIV tests conducted by untrained users in real world settings. *HIV Diagnostics Conference*; Atlanta, GA, USA; March 21– 24, 2016. E4.
15. Dong M, Regina R, Hlongwane S, Ghebremichael M, Wilson D, Dong K. Can laypersons in high prevalence South Africa perform an HIV self-test accurately? *International AIDS Conference*. Melbourne, VIC, Australia; July 20–25, 2014. WEPE034.
16. Gaydos CA, Hsieh YH, Harvey L, et al. Will patients "opt in" to perform their own rapid HIV test in the emergency department? *Ann Emerg Med* 2011; **58**: S74–S8.
17. Gaydos CA, Solis M, Hsieh YH, Jett-Goheen M, Nour S, Rothman RE. Use of tablet-based kiosks in the emergency department to guide patient HIV self-testing with a point-of-care oral fluid test. *Int J STD AIDS* 2013; **24**: 716–21.
18. Gras G, Le Bret P, Dailloux JF, et al. Low feasibiliy rate of self-testing with a finger-stick whole blood test. Top Antivir Med 2014; **22**: 512.
19. Kurth AE, Cleland CM, Chhun N, et al. Accuracy and acceptability of oral fluid HIV self-testing in a general adult population in Kenya. *AIDS Behav* 2016; **20**: 870–79.
20. Li YF, Wang YM, Zhang RR, et al. Analysis on accuracy and influencing factors of oral fluid-based rapid HIV self-testing among men who have sex with men. *Zhonghua Liu Xing Bing Xue Za Zhi* 2016; 37: 72–75 (in Chinese).
21. Nour S, Hsieh YH, Rothman RE, et al. Patients can accurately perform their own rapid HIV point-of-care test in the emergency department. *Point Care* 2012; **11**: 176–79.
22. Spielberg F, Camp S, Ramachandra E. HIV home self-testing: can it work? *National HIV Prevention Conference*; Atlanta, GA, USA; July 27–30, 2003. 03-A-1007-NHPC.
23. Mavedzenge SN, Sibanda E, Mavengere Y, et al. Supervised HIV self-testing to inform implementation and scale up of self-testing in Zimbabwe. *J Int AIDS Soc* 2015; **18**: 96.
24. Ng OT, Chow AL, Lee VJ, et al. Accuracy and user-acceptability of HIV self-testing using an oral fluid-based HIV rapid test. *PLoS One* 2012; **7**: e45168.
25. Summary of safety and effectiveness. Rockville, MD: *US Food and Drug Administration*, 2012.
https://www.fda.gov/downloads/BiologicsBloodVaccines/BloodBloodProducts/ApprovedProducts/PremarketApprovalsPMAs/UCM312534.pdf (accessed May 12,
2016).
